# Supplementary material for: Early human fetal lung atlas reveals the temporal dynamics of epithelial cell plasticity
Source: Nat Commun. 2024 Jul 13;15:5898. doi: 10.1038/s41467-024-50281-5 (PMC11246468; doi:10.1038/s41467-024-50281-5)
Supplement: Supplementary file 1 — Supplementary Information [file 41467_2024_50281_MOESM1_ESM.pdf]

Supplementary Information

Early human fetal lung atlas reveals the temporal dynamics of epithelial cell plasticity

Henry Quach[1,2*], **Spencer Farrell**[3*], Ming Jia Michael Wu[1], Kayshani Kanagarajah[1,2], Joseph Wai-Hin Leung[4], Xiaoqiao Xu[5], Prajkta Kallurkar[5], Andrei L. Turinsky[5], Christine E. Bear[6], Felix Ratjen[7], Brian Kalish[4,8-9], Sidhartha Goyal[3], Theo J. Moraes[7], Amy P. Wong[1,2]

[*] These authors contributed equally.

1. Program in Developmental and Stem Cell Biology, Hospital for Sick Children, 686 Bay Street, Toronto, Ontario M5G 0A4, Canada
2. Department of Laboratory Medicine & Pathobiology, University of Toronto, Toronto, Ontario M5G 1A8, Canada
3. Department of Physics, University of Toronto, Toronto, Ontario M5G 1A8, Canada
4. Program in Neurosciences and Mental Health, Hospital for Sick Children, 686 Bay Street, Toronto, Ontario M5G 0A4, Canada
5. Centre for Computational Medicine, Hospital for Sick Children, 686 Bay Street, Toronto, Ontario M5G 0A4, Canada
6. Program in Molecular Medicine, Hospital for Sick Children, 686 Bay Street, Toronto, Ontario M5G 0A4, Canada
7. Program in Translational Medicine, Hospital for Sick Children, 686 Bay Street, Toronto, Ontario M5G 0A4, Canada
8. Department of Molecular Genetics, University of Toronto, Toronto, Ontario M5G 1A8, Canada
9. Division of Neonatology, Department of Paediatrics, Hospital for Sick Children, Toronto, Ontario M5G 1L7, Canada

Supplementary Figures

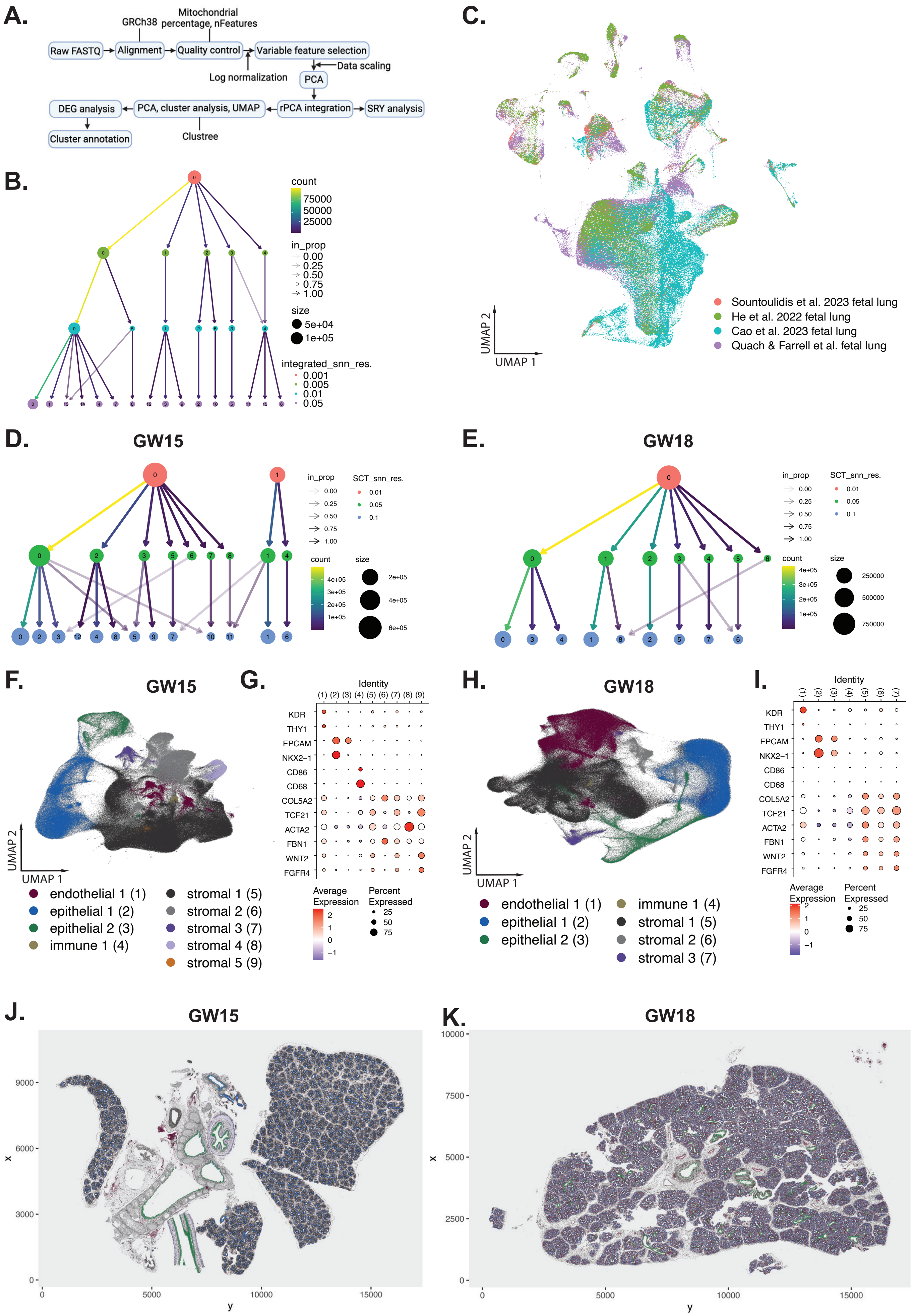

Supplementary Fig. 1: Analytical pipeline and characterization of the human fetal lung.
A: Computational pipeline for single-cell RNA sequencing analysis. Graphical image was created with Biorender.com.
B: Clustree analysis to determine the number of cell clusters. We selected the resolution in which the number of cell clusters would not yield "overlapping" subpopulations.
C: Integrated UMAP projections of all four datasets of human fetal lungs (He et al., 2022, Cao et al., 2023, Sountoulidis et al., 2023, and our dataset) with each dataset shown in different colours.
D, E: Clustree analysis performed on GW15 and GW18 Xenium dataset to determine clustering resolution of 0.05.
F, H: UMAP projection of 9 and 7 unique cell types from GW15 and GW18 Xenium dataset, respectively.
G, I: Dotplot of DEGs average expression from each cell type.
J, K: ImageDimPlot showing spatial distribution of cell type in GW15 and G18 fetal lung tissue. Cell types are coloured based on the legend in respective UMAPs.
Identity numbers in G and I indicate cell type as in F and H, respectively.
Source data are provided as a Source Data file.

A. Quach et al. qeuried to He et al. (2022)

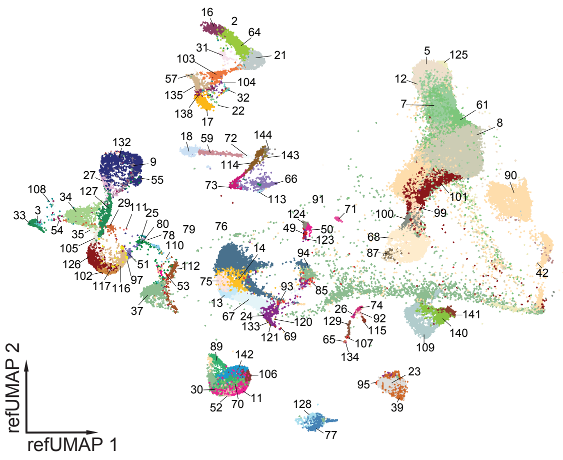

#	Var1	Freq
2	Activated NK	1
3	aDC 1	74
5	Adventitial fibro	2850
7	Airway fibro	7585
8	Alveolar fibro	32140
9	APOE+ Mφ1	181
11	Arterial endo	1677
12	ASPN+ chondrocyte	24
13	AT1	618
14	AT2	976
16	CD16+ NK	247
17	CD4 T	415
18	CD5- Mature B	283
21	CD56bright NK	1157
22	CD8 T	17
23	Ciliated	1749
24	Club	1521
25	CMP	6
26	COL20A1+ Schwann	2
27	CX3CR1+ Mφ	324
29	Cycling DC	85
30	Cycling definitive erythroblas	718
31	Cycling NK	96
32	Cycling T	58
33	DC1	347
34	DC2	732
35	DC3	84
37	Definitive erythrocyte	14374
39	Deuterosomal	1081
42	Early fibro	55
49	GHRL+ NE precursor	31
50	GHRL+ neuroendocrine	173
51	GMP	68
52	GRIA2+ arterial endo	459
53	HMOX1+ primitive erythrob	144
54	HSC	49
55	HSC/ELP	54
57	ILC3	324

#	Var1	Freq
59	Immature B	235
61	Interm fibro	8697
64	Intermediate NK	1125
65	KCNIP4+ neuron	4
66	Large pre-B	405
67	Late airway progenitor	1885
68	Late airway SMC	2632
69	Late basal	6
70	Late cap	2516
71	Late mesothelial	194
72	Late pre-B	74
73	Late pro-B	304
74	Late Schwann	385
75	Late stalk	243
76	Late tip	3916
77	Lymphatic endo	1539
78	Mast	511
79	Megakaryocyte	380
80	MEP	145
85	Mid airway progenitor	181
87	Mid airway SMC 2	63
89	Mid cap	2028
90	Mid fibro	19828
91	Mid mesothelial	2
92	Mid Schwann	46
93	Mid stalk	28
94	Mid tip	20
95	MUC16+ ciliated	7
97	Myelocyte-like	35
99	Myofibro 1	384
100	Myofibro 2	838
101	Myofibro 3	9687
102	Neutrophil	497
103	NKT1	358
104	NKT2	28
105	Non-cla. mono.	175
106	OMD+ endo	325
107	PCP4+ neuron	2

#	Var1	Freq
108	pDC	182
109	Pericyte	2074
110	Platelet	3
111	pre-pDC/DC5	6
112	Primitive erythrocyte	225
113	Pro-B	54
114	Pro-B/Pre-B transition	415
115	Proliferating Schwann	27
116	Promonocyte-like	435
117	Promyelocyte-like	97
120	Proximal secretory 2	21
121	Proximal secretory 3	19
123	Pulmonary NE precursor	39
124	Pulmonary neuroendocrine	336
125	Resting chondrocyte	571
126	S100A12-hi cla. mono.	1280
127	S100A12-lo cla. mono.	621
128	SCG3+ lymphatic endothelia	529
129	Schwann precursor	50
132	SPP1+ Mφ	4103
133	Squamous	13
134	SST+ neuron	2
135	Th17	358
138	Treg	104
140	Vascular SMC 1	600
141	Vascular SMC 2	476
142	Venous endo	1179
143	κ small pre-B	26
144	λ small pre-B	28

#	Not Detected
1	ACTC+ SMC
4	aDC 2
6	Aerocyte
10	APOE+ Mφ2
15	Basophil
19	CD5+ CCL22- mature B
20	CD5+ CCL22+ mature B
28	CXCL9+ Mφ
36	Definitive erythroblast
38	Definitive reticulocyte
40	Early airway progenitor
41	Early cap
43	Early mesothelial
44	Early Schwann
45	Early stalk
46	Early tip
47	Eosinophil
48	FGFBP2+ Neural progenitor
56	ILC2
58	ILCP
60	Interm chondrocyte
62	Interm neuroendocrine
63	Intermediate lymphatic endo
81	Mesenchymal 1
82	Mesenchymal 2
83	Mesenchymal 3
84	MFNG+ DBH+ neuron
86	Mid airway SMC 1
88	Mid basal
96	MUC5AC+ ASCL1+ progenitor
98	MYL4+ SMC
118	Proximal basal
119	Proximal secretory 1
122	Proximal secretory progenitors
130	SMG
131	SMG basal
136	TM4SF4+ CHODL+ neuron
137	TM4SF4+ PENK+ neuron
139	Tαβ Entry

B. Quach et al. qeuried to Sountoulidis et al. (2023)

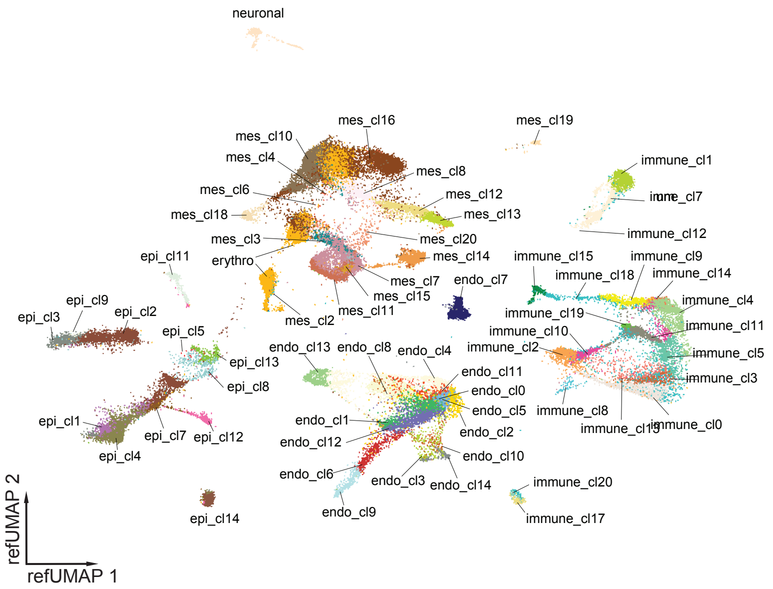

Var1	Detailed name	Freq
endo_cl0	Immature1	8
endo_cl1	Immature2	1438
endo_cl10	Proliferating1	240
endo_cl11	Immature3	450
endo_cl12	Immature arterial1	1429
endo_cl13	Venous	825
endo_cl14	Proliferating2	241
endo_cl2	Capillary	553
endo_cl3	Proliferating3	351
endo_cl4	Immature venous	1121
endo_cl5	Immature4	456
endo_cl6	Immature arterial2	467
endo_cl7	Lymphatic	2101
endo_cl8	Bronchial	1374
endo_cl9	Arterial	468
epi_cl1	Intermediate	555
epi_cl11	NE1	253
epi_cl12	NE2	456
epi_cl13	Proliferating2	238
epi_cl14	Ciliated	2723
epi_cl2	SOX9 high ETV5 med distal	3950
epi_cl3	CTGF high distal	1539
epi_cl4	Prox. progenitor2	3154
epi_cl5	Proliferating3	36
epi_cl7	NE progenitor	470
epi_cl8	Proliferating1	702
epi_cl9	SFTPC high distal	90
erythro	erythro	41662
immune_cl0	Immature macrophage2	1521
immune_cl1	Natural killer	2434
immune_cl10	Immature monocyte	796
immune_cl11	Dendritic	920

Var1	Detailed name	Freq
immune_cl12	ILC3	178
immune_cl13	Myloid progenitor3	333
immune_cl14	Monocyte	110
immune_cl15	B cell	1420
immune_cl17	Megakaryocyte2	363
immune_cl18	Mast/basophil	1874
immune_cl19	Conventional dendritic	394
immune_cl2	Immature macrophage1	1511
immune_cl20	Megakaryocyte1	65
immune_cl3	Myeloid progenitor2	315
immune_cl4	Immature monocyte2	1304
immune_cl5	Migrating dendritic	717
immune_cl7	Lymphoid progenitor	1683
immune_cl8	Proliferating myeloid	76
immune_cl9	Neutrophils	696
mes_cl10	Adv. fibro	19840
mes_cl11	Proliferating2	4022
mes_cl12	Imm ASM2	1672
mes_cl13	ASM	2088
mes_cl14	Pericyte	3091
mes_cl15	Proliferating1	544
mes_cl16	AF	14157
mes_cl18	Chondroblast	497
mes_cl19	Mesothelial	200
mes_cl2	Immature2	17
mes_cl20	Prol. ASM	270
mes_cl3	Proliferating1	1267
mes_cl4	Imm. AF1	135
mes_cl6	Immature5	3615
mes_cl7	Proliferating3	3215
mes_cl8	Imm. ASM1	5172
neuronal	neuronal	518

Not detected	
Var1	Detailed name
epi_cl6	Prox. progenitor1
epi_cl10	SOX9 high ETV5 hi distal
epi_cl0	Prox. secretory
mes_cl0	Imamture 1
mes_cl1	Immature 4
mes_cl5	Imm. AF2

Not detected	
Var1	Detailed name
mes_cl9	Imm. adv. fibro
immune_cl6	Myeloid progenitor1
immune_cl21	ILC2
immune_cl16	Macrophage
mes_cl17	Immature 3

C. Quach et al. qeuried to Cao et al. (2023)

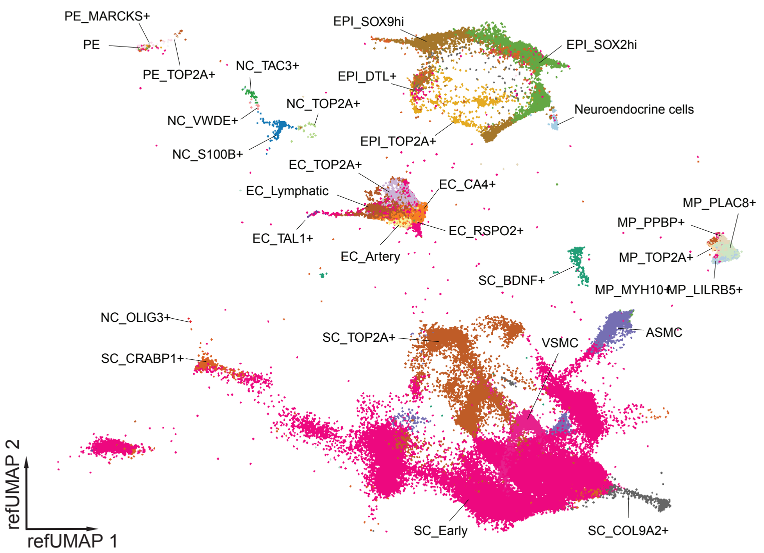

Cell Type	Freq
ASMC	2773
EC_Artery	660
EC_CA4+	6739
EC_Lymphatic	3123
EC_RSPO2+	68
EC_TAL1+	13
EC_TOP2A+	1635
EPI_DTL+	89
EPI_SOX2hi	6866
EPI_SOX9hi	3957
EPI_TOP2A+	543
MP_LILRB5+	2884
MP_MYH10+	35
MP_PLAC8+	8369
MP_PPBP+	5137
MP_TOP2A+	110
NC_OLIG3+	3
NC_S100B+	441

Cell Type	Freq
NC_TAC3+	31
NC_TOP2A+	31
NC_VWDE+	11
Neuroendocrine cells	520
PE	425
PE_MARCKS+	37
PE_TOP2A+	12
SC_BDNF+	302
SC_COL9A2+	751
SC_CRABP1+	1395
SC_Early	87340
SC_TOP2A+	7001
VSMC	3079

Undetected Cells
MP_HSPA6+
MP_PDGFRA+

Supplementary Fig. 2: Comparison to recently published fetal lung datasets.
A: MapQuery analysis using He et al. (2022) dataset as the reference map to identify cell subtypes common with our dataset.
B: MapQuery analysis using Sountoulidis et al. (2023) dataset as the reference map to identify cell subtypes common with our dataset.
C: MapQuery analysis using Cao et al. (2023) dataset as the reference map to identify cell subtypes common with our dataset.

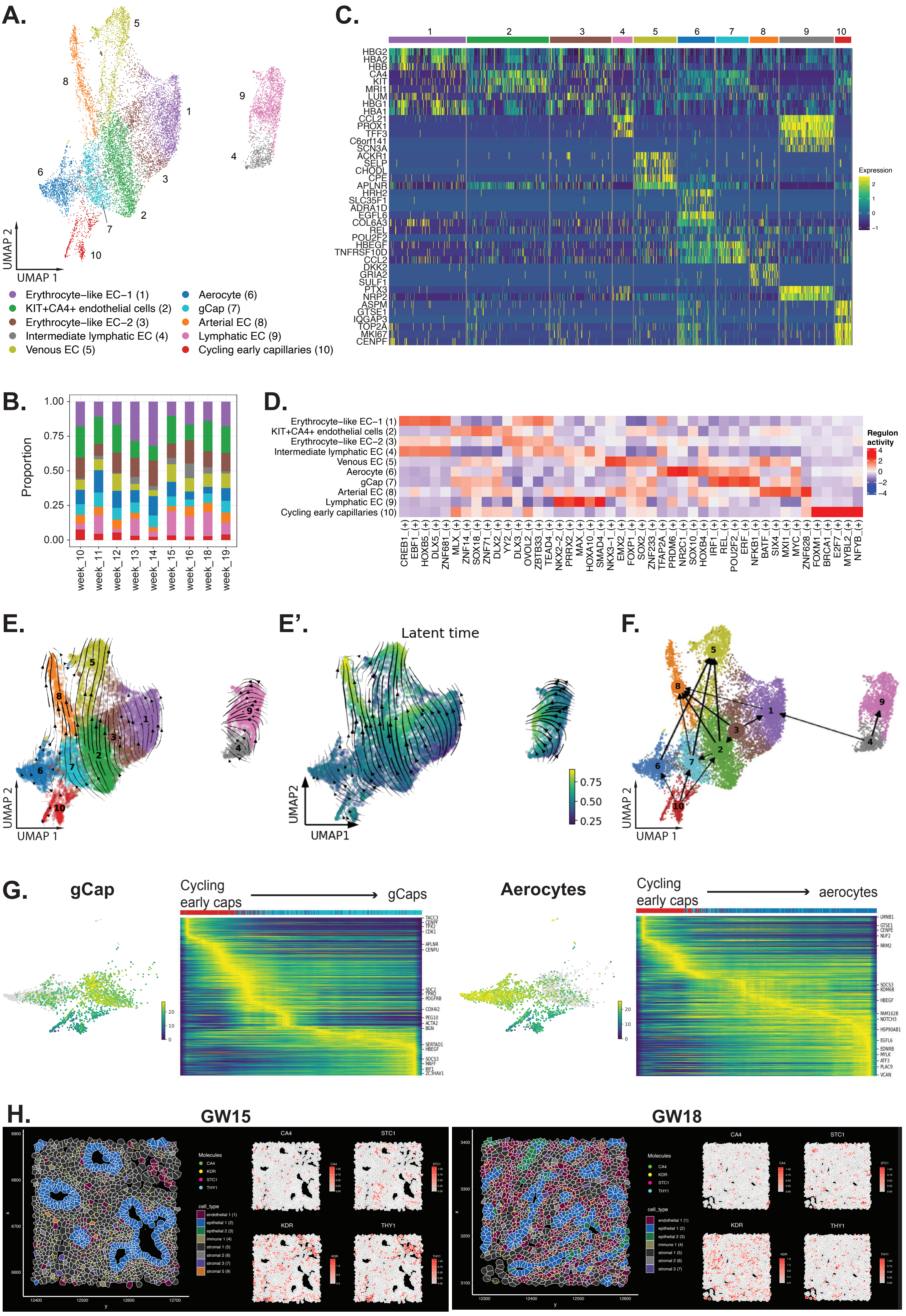
A.
C.
B.
D.
E.
E'.
Latent time
F.
G.
gCap
Cycling early caps
gCaps
Aerocytes
Cycling early caps
aerocytes
H.
GW15
GW18
UMAP 1
UMAP 2
Erythrocyte–like EC–1 (1)
KIT+CA4+ endothelial cells (2)
Erythrocyte–like EC–2 (3)
Intermediate lymphatic EC (4)
Venous EC (5)
Aerocyte (6)
gCap (7)
Arterial EC (8)
Lymphatic EC (9)
Cycling early capillaries (10)
Expression
Proportion
Regulon activity
CA4
STC1
KDR
THY1

Supplementary Fig. 3: Co-development of the fetal pulmonary endothelium.
A: UMAP visualization of the fetal endothelial subtypes.
B: Gene expression heatmap of DEGs representing each endothelial subtype.
C: Proportion of the endothelial cell types across GW.
D: Dotplot measuring regulon activity of the top differentially expressed transcription factors (TF) genes based on regulon specificity score (RSS) via *SCENIC.*
E: UMAP projection of inferred LatentVelo velocities (arrows) and **E':** LatentVelo latent times. UMAP coloured by pseudotime, dark blue to yellow.
F: PAGA using LatentVelo velocities (arrows). Line thickness indicates inferred transition strength.
G: Slingshot trajectory analysis using a root at Cycling early capillaries, as identified by LatentVelo. Trajectories to gCaps and aerocytes are identified. Trajectory heatmaps indicate the progression of cell types and significantly varying genes along the trajectories.
H: Spatial localization of *STC1* and *THY1* associated with gCaps and aerocytes respectively in GW 15 (left) and 18 (right) tissues. Endothelial markers *CA4* and *KDR* is also shown for additional context.
Colors in B, E, and F indicates cell type as in A.
Source data are provided as a Source Data file.

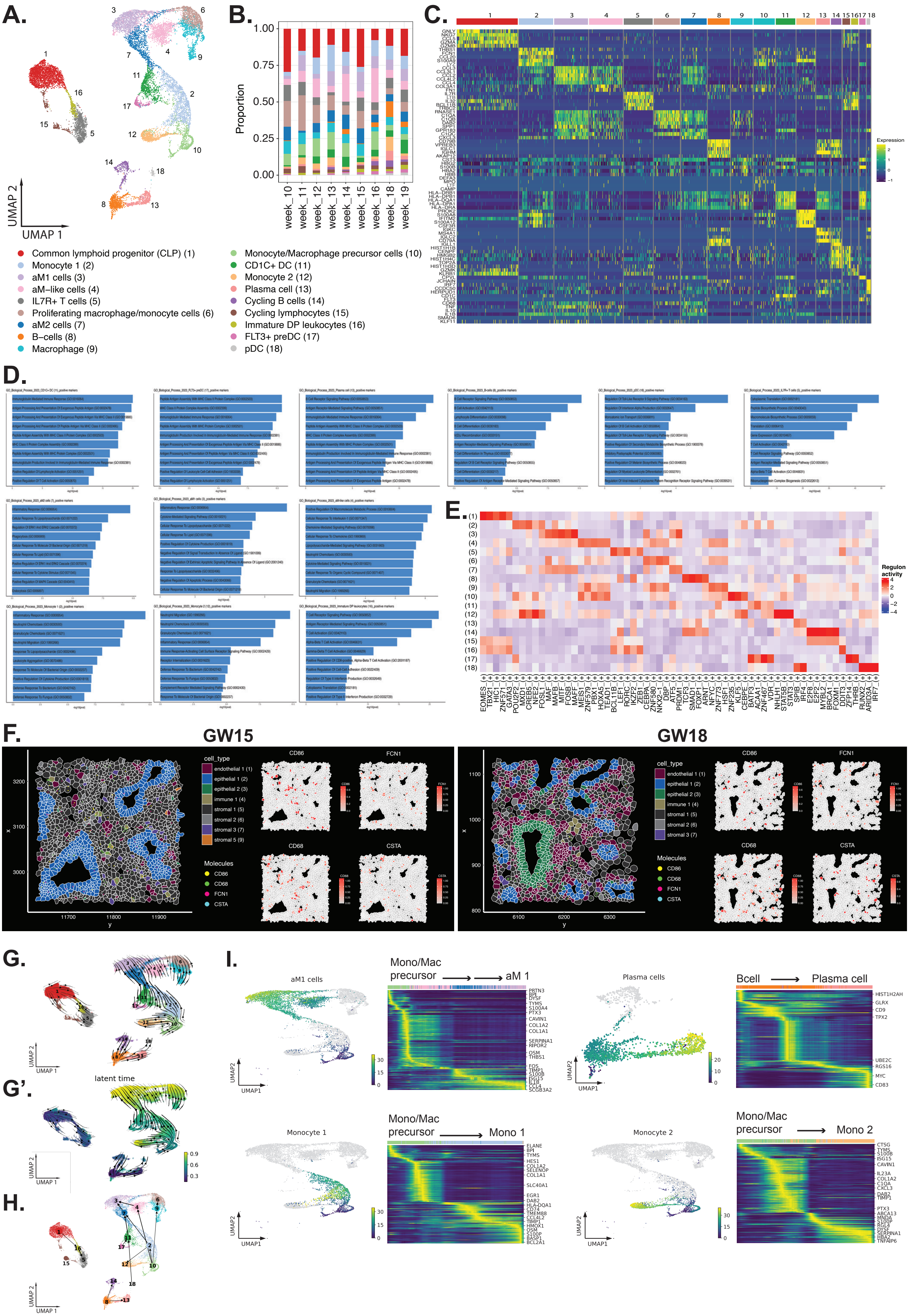
A.
UMAP 2
UMAP 1
Common lymphoid progenitor (CLP) (1)
Monocyte 1 (2)
aM1 cells (3)
aM–like cells (4)
IL7R+ T cells (5)
Proliferating macrophage/monocyte cells (6)
aM2 cells (7)
B–cells (8)
Macrophage (9)
Monocyte/Macrophage precursor cells (10)
CD1C+ DC (11)
Monocyte 2 (12)
Plasma cell (13)
Cycling B cells (14)
Cycling lymphocytes (15)
Immature DP leukocytes (16)
FLT3+ preDC (17)
pDC (18)
B.
Proportion
1.00
0.75
0.50
0.25
0.00
week_10
week_11
week_12
week_13
week_14
week_15
week_16
week_18
week_19
C.
Expression
D.
E.
Regulon activity
F.
GW15
GW18
cell_type
endothelial 1 (1)
epithelial 1 (2)
epithelial 2 (3)
immune 1 (4)
stromal 1 (5)
stromal 2 (6)
stromal 3 (7)
stromal 5 (9)
Molecules
CD86
CD68
FCN1
CSTA
G.
G'.
latent time
H.
I.
aM1 cells
Mono/Mac precursor → aM 1
Plasma cells
Bcell → Plasma cell
Monocyte 1
Mono/Mac precursor → Mono 1
Monocyte 2
Mono/Mac precursor → Mono 2

Supplementary Fig. 4: Novel alveolar macrophage lineage trajectories identified in the fetal lung.

A: UMAP visualization of the fetal immune cell subtypes.
B: Proportion of the immune cell types across GW.
C: Gene expression heatmap of DEGs representing each immune cell subtype.
D: Gene ontology terms enriched for each of the major immune cell subtypes.
E: Dotplot measuring regulon activity of the top differentially expressed transcription factors (TF) genes based on regulon specificity score (RSS) via *SCENIC.* Numbers indicate cell type as in A.
F: Xenium spatial analysis of *CD86, CD68, FCN1,* and *CSTA* in GW15 (left) and 18 (right) tissues.
G: UMAP projection of inferred LatentVelo velocities (arrows) and **G':** LatentVelo latent times. UMAP coloured by pseudotime, dark blue to yellow.
H: PAGA using LatentVelo velocities (arrows). Line thickness indicates inferred transition strength.
I: Slingshot trajectory analysis using a root at Monocyte/Macrophage precursor cells, as identified by LatentVelo. Trajectories to aM1, and Monocyte 1 and 2 are identified. Using a root at B cells, a trajectory is inferred to Plasma cells. Trajectory heatmaps indicate the progression of cell types and significantly varying genes along the trajectories.
Colors in B, top bar in C, G, H indicate cell type as in A.
Numbers in C, and E indicate cell type as in A.
Source data are provided as a Source Data file.

A. Sikkema et al. (2023) data queried to Quach et al. data

Query transferred labels

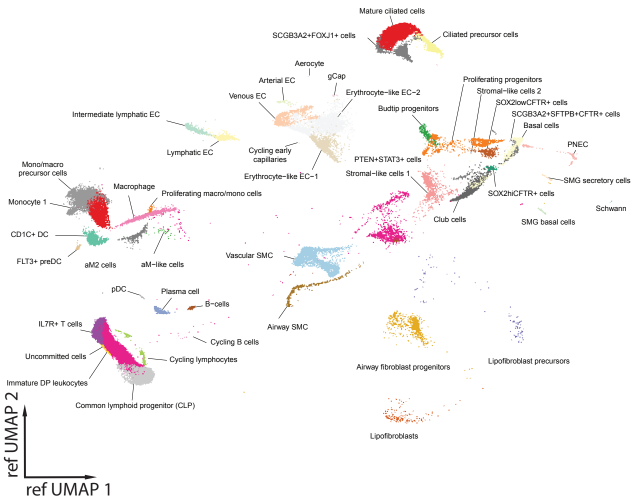

Cell Type	# of Cells
Aerocyte	1
Airway fibroblast progenitors	662
Airway SMC	312
aM-like cells	32
aM2 cells	264
Arterial EC	72
B-cells	96
Basal cells	13845
Budtip progenitors	207
CD1C+ DC	782
Ciliated precursor cells	1097
Club cells	7206
Common lymphoid progenitor (CLP)	13149
Cycling B cells	6
Cycling early capillaries	5
Cycling lymphocytes	262
Erythrocyte-like EC-1	1025
Erythrocyte-like EC-2	2816
FLT3+ preDC	70
gCap	8

Cell Type	# of Cells
IL7R+ T cells	8368
Immature DP leukocytes	260
Intermediate lymphatic EC	693
Lipofibroblast precursors	92
Lipofibroblasts	146
Lymphatic EC	735
Macrophage	1219
Mature ciliated cells	4647
Monocyte 1	4257
Monocyte/macrophage precursor cells	5017
pDC	126
Plasma cell	689
PNEC	188
Proliferating macrophage/mono cells	51
Proliferating progenitors	1125
PTEN+STAT3+ cells	61
SCGB3A2+FOXJ1+ cells	1934
SCGB3A2+SFTPB+CFTR+ cells	184
Schwann	3
SMG basal cells	78

Cell Type	# of Cells
SMG secretory cells	20
SOX2highCFTR+ cells	66
SOX2lowCFTR+ cells	266
Stromal-like cells 1	721
Stromal-like cells 2	3
Uncommitted cells	11137
Vascular SMC	3295
Venous EC	957

Unmapped Cell Clusters
aM1 cells
Chondrocyte
Cycling fibroblasts
Tip cells
Early fibroblasts
KIT+CA4+ endothelial cells
Monocyte 2
NKX2-1+SOX9+CFTR+ cells
NRGN+ cells
Unknown stromal cells

B. Quach et al. data queried to Sikkema et al. (2023) data

Query transferred labels

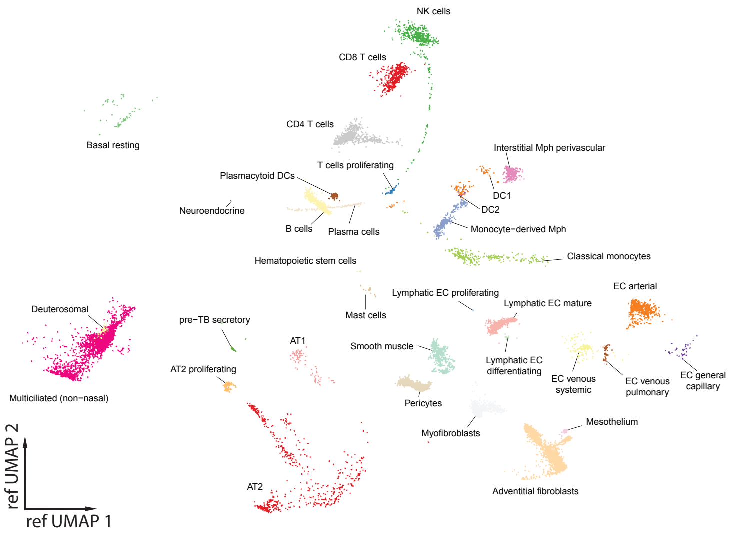

Cell Type	# of cells
Adventitial fibroblasts	2848
AT1	73
AT2	522
AT2 proliferating	109
B cells	463
Basal resting	54
CD4 T cells	636
CD8 T cells	398
Classical monocytes	244
DC1	11
DC2	88
Deuterosomal	90
EC arterial	501
EC general capillary	32
EC venous pulmonary	42
EC venous systemic	147
Hematopoietic stem cells	7
Interstitial Mph perivascular	292
Lymphatic EC differentiating	11
Lymphatic EC mature	781
Lymphatic EC proliferating	3

Cell Type	# of cells
Mast cells	13
Mesothelium	106
Monocyte-derived Mph	260
Multiciliated (non-nasal)	2466
Myofibroblasts	1281
Neuroendocrine	1
NK cells	418
Pericytes	1021
Plasma cells	102
Plasmacytoid DCs	97
pre-TB secretory	19
Smooth muscle	498
T cells proliferating	43

Unmapped Cell Clusters
Alveolar fibroblasts
Alveolar macrophages
Alveolar Mph CCL3+
Alveolar Mph MT-positive
Alveolar Mph proliferating
AT0

Unmapped Cell Clusters
Club (nasal)
Club (non-nasal)
EC aerocyte capillary
Goblet (bronchial)
Goblet (nasal)
Goblet (subsegmental)
Hillock-like
Ionocyte
Migratory DCs
Multiciliated (nasal)
Non-classical monocytes
Peribronchial fibroblasts
SM activated stress response
SMG duct
SMG mucous
SMG serous (bronchial)
SMG serous (nasal)
Smooth muscle FAM83D+
Subpleural fibroblasts
Suprabasal
Tuft

C. Negretti et al. (2021) data queried to Quach et al. data

Query transferred labels

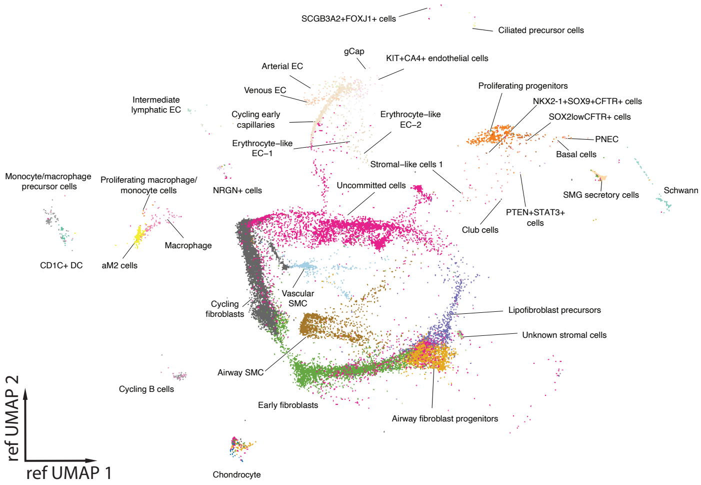

Cell Type	# of Cells
Airway fibroblast progenitors	1783
Airway SMC	1192
aM2 cells	140
Arterial EC	25
Basal cells	4
CD1C+ DC	31
Chondrocyte	31
Ciliated precursor cells	5
Club cells	2
Cycling B cells	23
Cycling early capillaries	720
Cycling fibroblasts	3895
Early fibroblasts	2898
Erythrocyte-like EC-1	85
Erythrocyte-like EC-2	83
gCap	4
Intermediate lymphatic EC	13
KIT+CA4+ endothelial cells	50
Lipofibroblast precursors	660
Macrophage	76
Monocyte/macrophage precursor cells	378

Cell Type	# of Cells
NKX2-1+SOX9+CFTR+ cells	3
NRGN+ cells	16
PNEC	10
Proliferating macrophage/ monocyte cells	6
Proliferating progenitors	342
PTEN+STAT3+ cells	4
SCGB3A2+FOXJ1+ cells	3
Schwann	64
SMG secretory cells	355
SOX2lowCFTR+ cells	39
Stromal-like cells 1	45
Uncommitted cells	5709
Unknown stromal cells	78
Vascular SMC	345
Venous EC	89

Unmapped Cell Clusters
aM1 cells
SCGB3A2+SFTPB+CFTR+ cells
SOX2highCFTR+ cells
Lipofibroblasts
Budtip progenitors
Mature ciliated cells
Tip cells
Stromal-like cells 2
SMG basal cells
Aerocyte
Lymphatic EC
Common lymphoid progenitor (CLP)
Monocyte 1
aM-like cells
IL7R+ T cells
B-cells
Monocyte 2
Plasma cell
Cycling lymphocytes
Immature DP leukocytes
FLT3+ preDC
pDC

Supplementary Fig. 5: Comparison of our dataset with existing human adult lung and mouse embryonic lung single cell RNA sequencing datasets.

A: MapQuery analysis using our dataset as the reference map to identify cell types common in adult lung tissues. Adult lung dataset was obtained from Sikkema et al., 2023.

B: Reverse query analysis using the HCA cell atlas (Sikkema et al., 2023) as the reference map to identify cell types common to our dataset.

C: MapQuery analysis using our dataset as the reference map to identify cell subtypes common in mouse embryonic lung tissues. Mouse embryonic lung (e12 and e15). dataset was obtained from Negretti et al. 2021. Reverse query analysis was not performed as the Negretti et al., 2021 data set did not include cell type labeling which limited our ability to perform cell-to-cell comparisons.

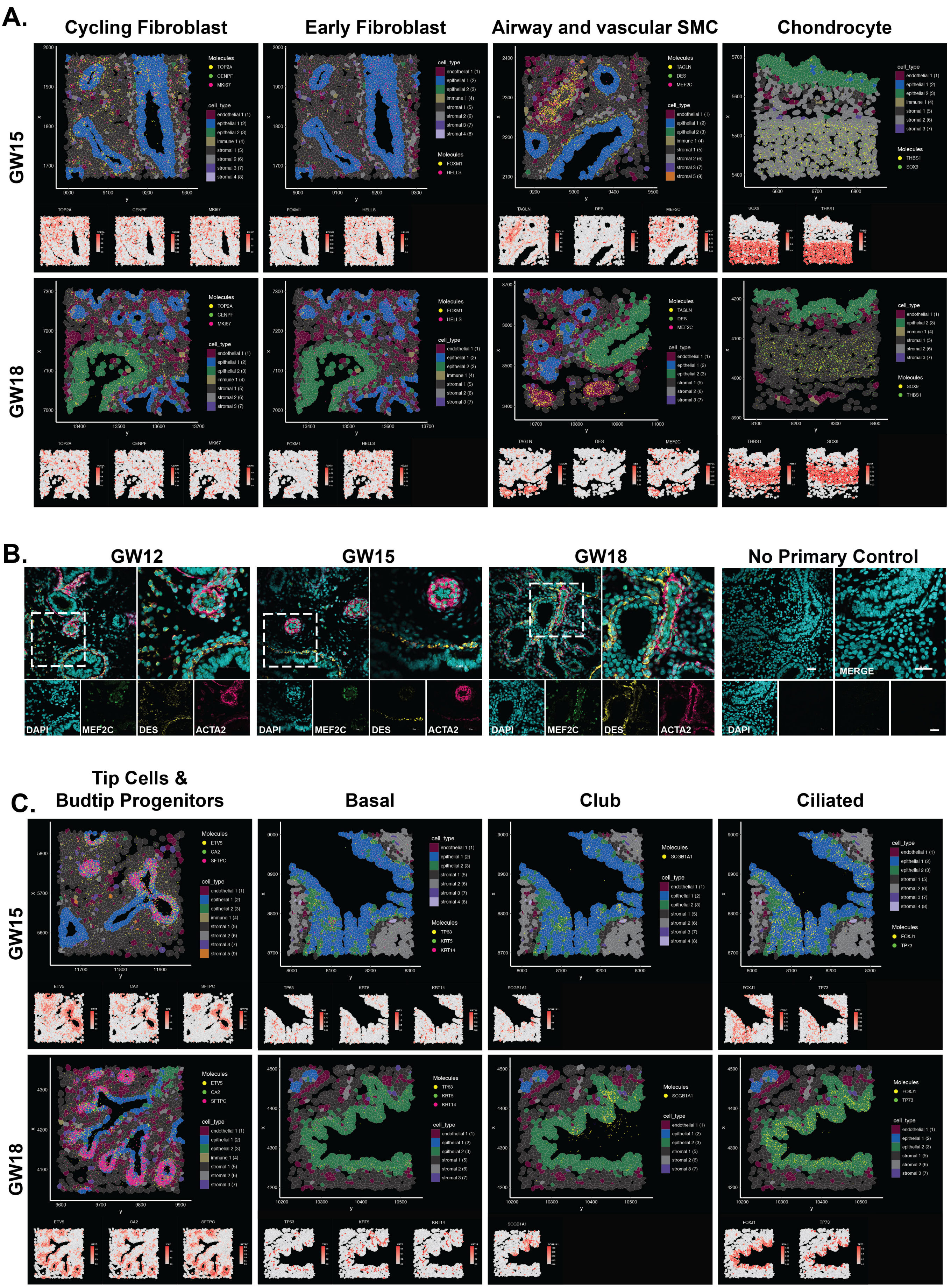
A.
Cycling Fibroblast
Early Fibroblast
Airway and vascular SMC
Chondrocyte
GW15
GW18
B.
GW12
GW15
GW18
No Primary Control
DAPI
MEF2C
DES
ACTA2
MERGE
C.
Tip Cells &
Budtip Progenitors
Basal
Club
Ciliated
GW15
GW18

Supplementary Fig. 6: Spatial transcriptomic of the fetal stromal cell compartment.
A: High resolution spatial plot of top DEGs associated with cycling fibroblasts, early fibroblasts, airway and vascular SMC and chondrocytes.
B: Immunofluorescence of MEF2C (vascular SMC, green), DES (airway SMC, yellow) and ACTA2 (pan-SMC, magenta) in GW12, 15 and 18 fetal lung tissue illustrates regions with both blood vessels and airways, highlighted by white hashed squares. At least 4 representative images were captured and analyzed for each gestational timepoint. Adjacent images are higher magnification views of the highlighted areas of interest.
C: High resolution spatial plot of top DEGs associated with budtip, tip, basal, club and ciliated cells.

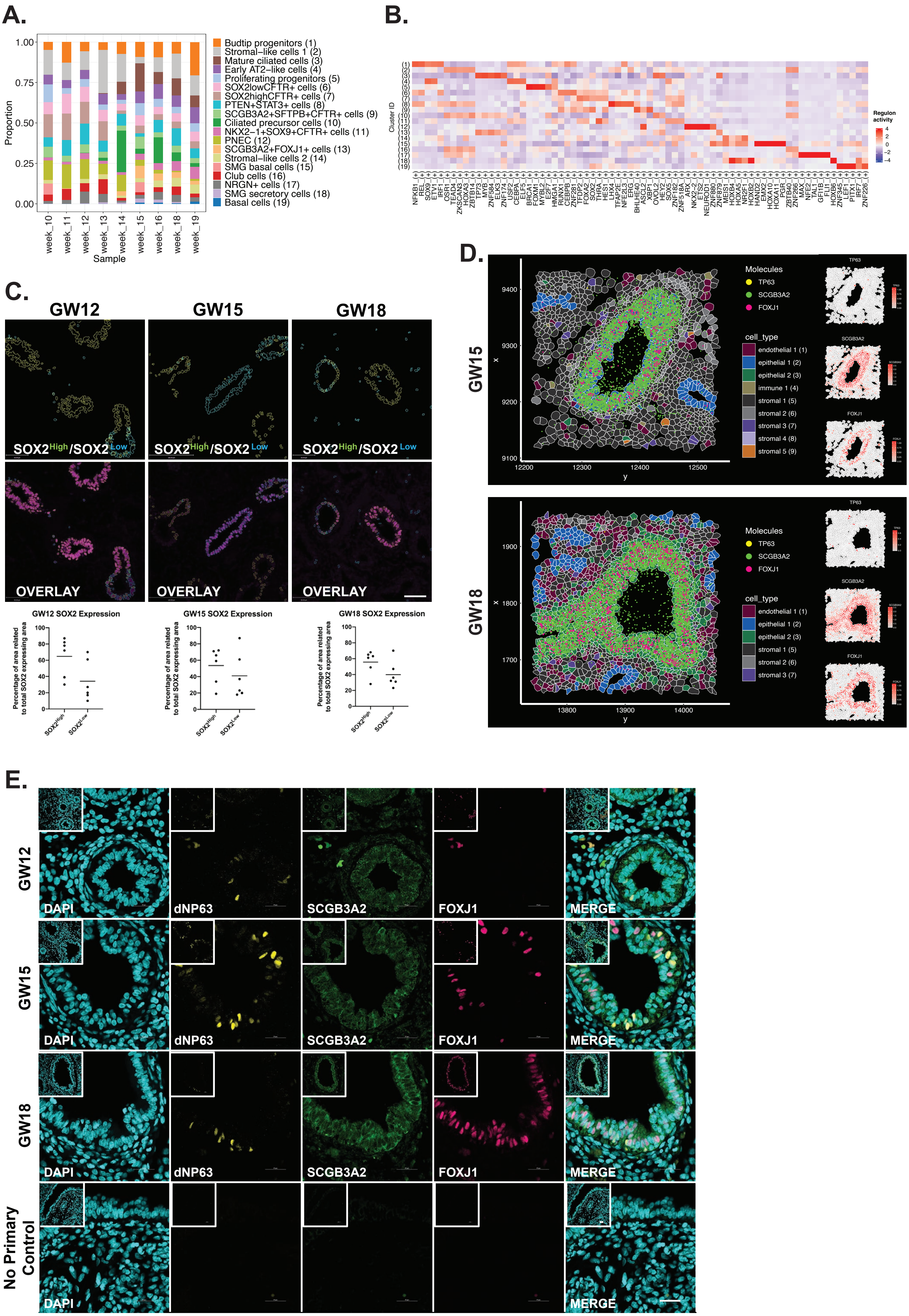
A.
B.
C.
D.
E.
Proportion
Sample
week_10
week_11
week_12
week_13
week_14
week_15
week_16
week_18
week_19
Budtip progenitors (1)
Stromal−like cells 1 (2)
Mature ciliated cells (3)
Early AT2−like cells (4)
Proliferating progenitors (5)
SOX2lowCFTR+ cells (6)
SOX2highCFTR+ cells (7)
PTEN+STAT3+ cells (8)
SCGB3A2+SFTPB+CFTR+ cells (9)
Ciliated precursor cells (10)
NKX2−1+SOX9+CFTR+ cells (11)
PNEC (12)
SCGB3A2+FOXJ1+ cells (13)
Stromal−like cells 2 (14)
SMG basal cells (15)
Club cells (16)
NRGN+ cells (17)
SMG secretory cells (18)
Basal cells (19)
Cluster ID
Regulon activity
GW12
GW15
GW18
SOX2 High /SOX2 Low
OVERLAY
GW12 SOX2 Expression
GW15 SOX2 Expression
GW18 SOX2 Expression
Percentage of area related to total SOX2 expressing area
SOX2 High
SOX2 Low
Molecules
TP63
SCGB3A2
FOXJ1
cell_type
endothelial 1 (1)
epithelial 1 (2)
epithelial 2 (3)
immune 1 (4)
stromal 1 (5)
stromal 2 (6)
stromal 3 (7)
stromal 4 (8)
stromal 5 (9)
DAPI
dNP63
SCGB3A2
FOXJ1
MERGE
No Primary Control

Supplementary Fig. 7: Characterization of the developing fetal lung epithelium.
A: Proportion of the epithelial cell subtypes across GW.
B: Dotplot measuring regulon activity of the top differentially expressed transcription factors (TF) genes based on regulon specificity score (RSS) via SCENIC.
C: Quantification of $SOX2^{high}$ and $SOX2^{low}$ in the developing airways. Bar represents average percentage of area segmented as either $SOX2^{high}$ or $SOX2^{low}$ relative to total SOX2 expression in GW12, 15 and 18 tissue. N=6 images per group. Scale bar = 50 microns.
D: High resolution Xenium spatial plots of *TP63, SCGB3A2* and *FOXJ1* in GW15 and GW18 tissue.
E: Immunofluorescence of deltaNP63 (basal cell, yellow), SCGB3A2 (secretory cell, green), and FOXJ1 (ciliated cell, magenta) in GW12, 15 and 18 fetal lung tissue. Insets are low magnification images of area of interest. At least 4 representative images were captured and analyzed for each gestational timepoint. Bottom row is the negative (no primary, secondary antibodies only) controls. DAPI marks all nuclei. Scale bar = 25 microns.
Cluster ID numbers in B indicates cell type as in A.
Source data are provided as a Source Data file.

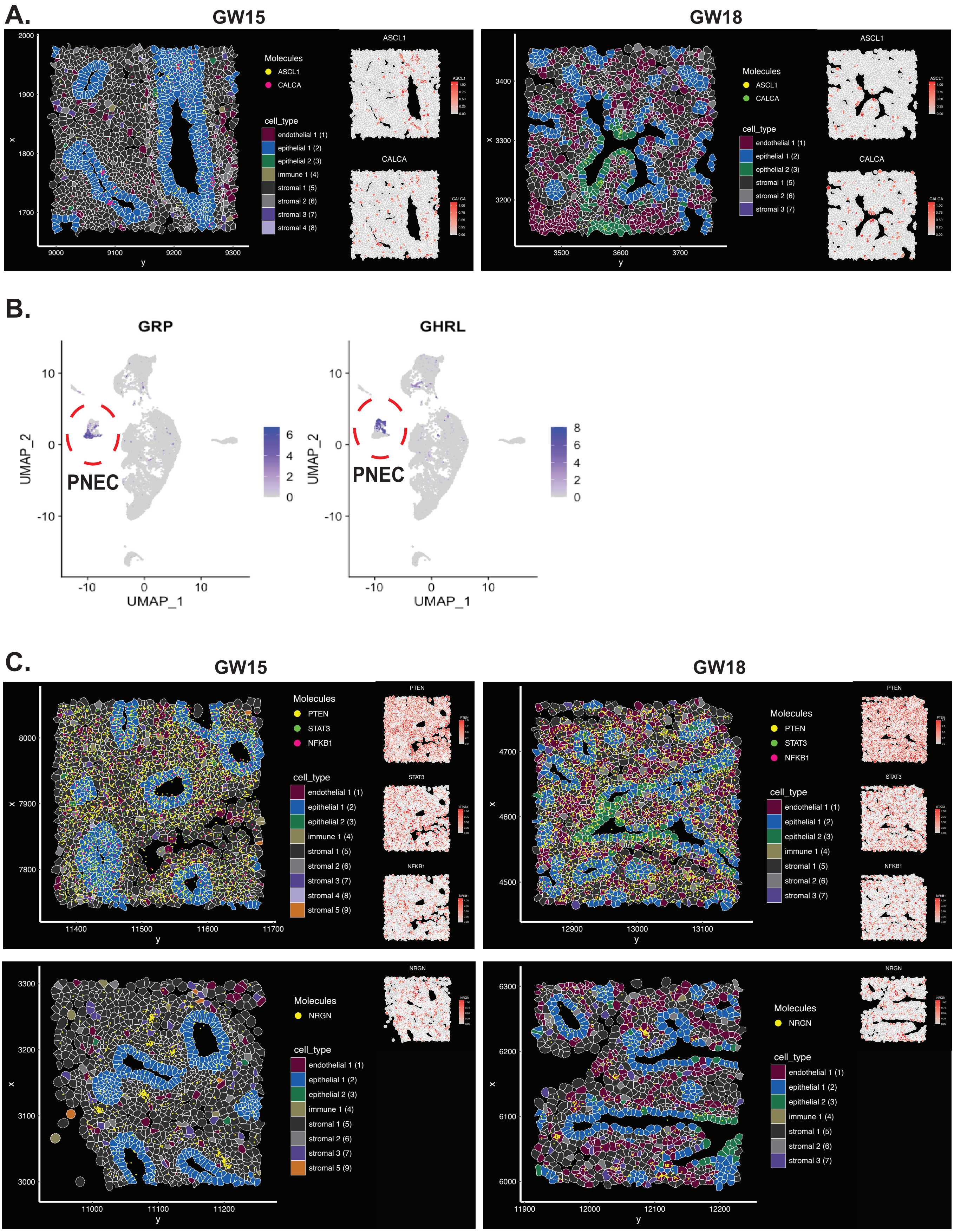
A.
GW15
GW18
Molecules
ASCL1
CALCA
cell_type
endothelial 1 (1)
epithelial 1 (2)
epithelial 2 (3)
immune 1 (4)
stromal 1 (5)
stromal 2 (6)
stromal 3 (7)
stromal 4 (8)
x
y
B.
GRP
GHRL
PNEC
UMAP_1
UMAP_2
C.
GW15
GW18
Molecules
PTEN
STAT3
NFKB1
NRGN
cell_type
endothelial 1 (1)
epithelial 1 (2)
epithelial 2 (3)
immune 1 (4)
stromal 1 (5)
stromal 2 (6)
stromal 3 (7)
stromal 4 (8)
stromal 5 (9)

Supplementary Fig. 8: Spatial determination of epithelial subtypes in the fetal lungs and identification of PNEC subsets.
A: High resolution Xenium spatial plots of *ASCL1* and *CALCA* in the developing airway epithelium.
B: UMAP feature plots for *GRP* and *GHRL* demarcating two PNEC populations also identified in previous datasets.
C: High resolution Xenium spatial plots of DEGs associated with *PTEN+STAT3+* and *NRGN+* cells.

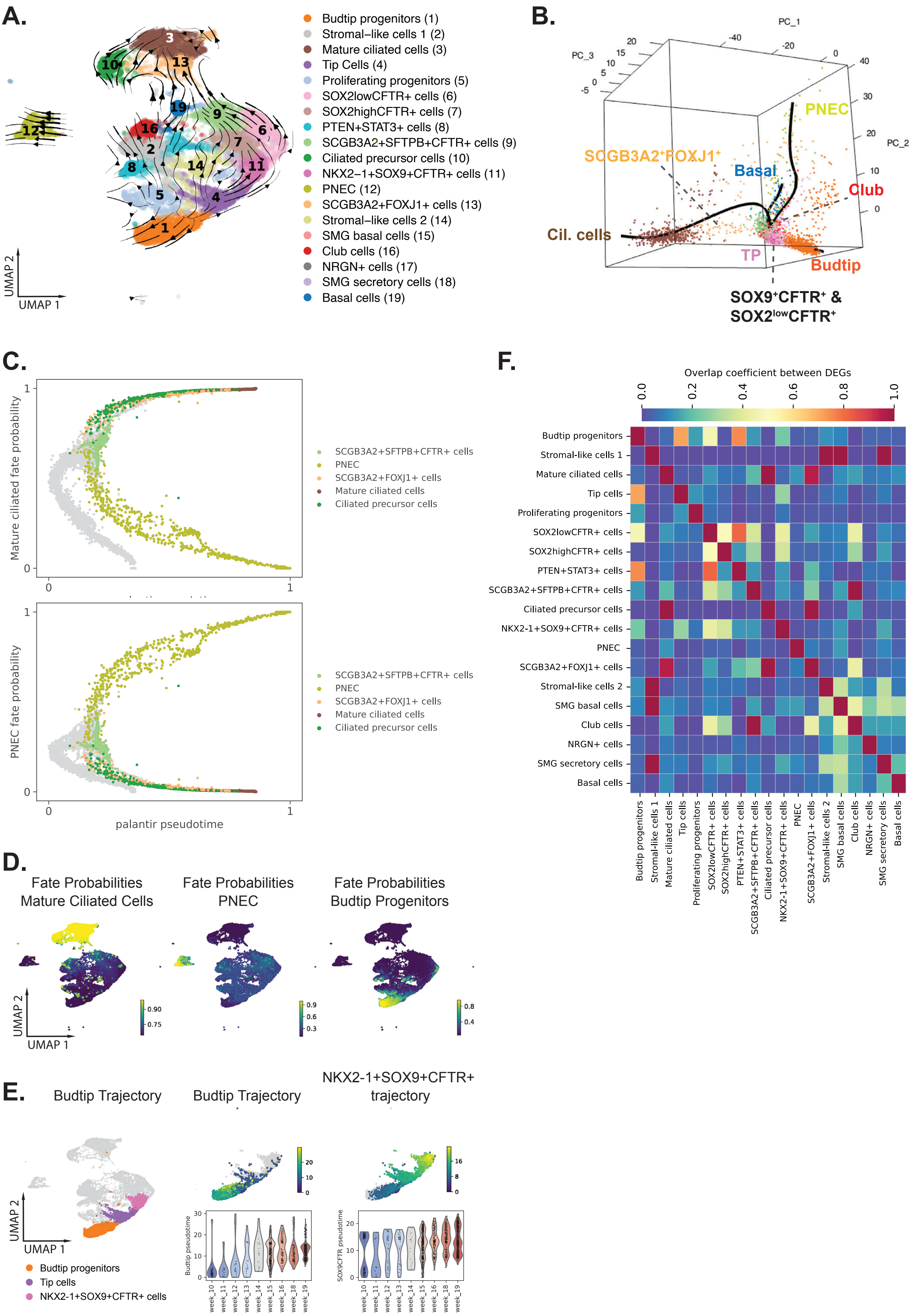
A.
Budtip progenitors (1)
Stromal–like cells 1 (2)
Mature ciliated cells (3)
Tip Cells (4)
Proliferating progenitors (5)
SOX2lowCFTR+ cells (6)
SOX2highCFTR+ cells (7)
PTEN+STAT3+ cells (8)
SCGB3A2+SFTPB+CFTR+ cells (9)
Ciliated precursor cells (10)
NKX2–1+SOX9+CFTR+ cells (11)
PNEC (12)
SCGB3A2+FOXJ1+ cells (13)
Stromal–like cells 2 (14)
SMG basal cells (15)
Club cells (16)
NRGN+ cells (17)
SMG secretory cells (18)
Basal cells (19)
UMAP 2
UMAP 1
B.
PC_1
PC_2
PC_3
PNEC
SCGB3A2+FOXJ1+
Basal
Club
Cil. cells
TP
Budtip
SOX9+CFTR+ &
SOX2lowCFTR+
C.
Mature ciliated fate probability
PNEC fate probability
palantir pseudotime
SCGB3A2+SFTPB+CFTR+ cells
PNEC
SCGB3A2+FOXJ1+ cells
Mature ciliated cells
Ciliated precursor cells
D.
Fate Probabilities
Mature Ciliated Cells
Fate Probabilities
PNEC
Fate Probabilities
Budtip Progenitors
E.
Budtip Trajectory
Budtip Trajectory
NKX2-1+SOX9+CFTR+
trajectory
Budtip progenitors
Tip cells
NKX2-1+SOX9+CFTR+ cells
Budtip pseudotime
SOX9CFTR pseudotime
F.
Overlap coefficient between DEGs
Budtip progenitors
Stromal-like cells 1
Mature ciliated cells
Tip cells
Proliferating progenitors
SOX2lowCFTR+ cells
SOX2highCFTR+ cells
PTEN+STAT3+ cells
SCGB3A2+SFTPB+CFTR+ cells
Ciliated precursor cells
NKX2-1+SOX9+CFTR+ cells
PNEC
SCGB3A2+FOXJ1+ cells
Stromal-like cells 2
SMG basal cells
Club cells
NRGN+ cells
SMG secretory cells
Basal cells

Supplementary Fig. 9: Validation of LatentVelo trajectories and analysis of distal trajectories.

A: Palantir was used to infer pseudotimes (arrows), starting with a root at GW10 budtip cells. CellRank was used to infer a transition matrix between cells, and transitions were projected onto the UMAP (arrows). Results largely agree with LatentVelo (Figure 6A), except for a clearer distinction of the terminal budtip state. The same terminal states at PNEC, mature ciliated cells, and later GW budtip cells were observed.

B: Slingshot analysis of late stage (GW 17-19) epithelia using budtip as the starting cluster and projected onto 3D PCA plot. A trifurcation appears from the TP cells towards PNEC, mature ciliated cells, and basal cells.

C: Mature ciliated cells and PNEC fate probabilities vs Palantir pseudotime through TP cells. TP cells were situated in the middle near 0.5, indicating their involvement in both lineages.

D: Fate probabilities for the 3 terminal states mature ciliated, PNEC and budtip terminal states. Note that budtip cells are increasing and our analysis has identified these as "terminal" states (yellow) at the end of our developmental time point.

E: Analysis of the budtip terminal state with Slingshot using an early GW root. A bifurcation going towards the late GW budtip terminal state, or towards tip cells and *NKX2-1+SOX9+CFTR+* cells was observed demonstrating the connection between these three cell types. Notably, there was a temporal increase in budtip and *NKX2-1+SOX9+CFTR+* cells.

F: DEG overlap between epithelial cell types.

Source data are provided as a Source Data file.

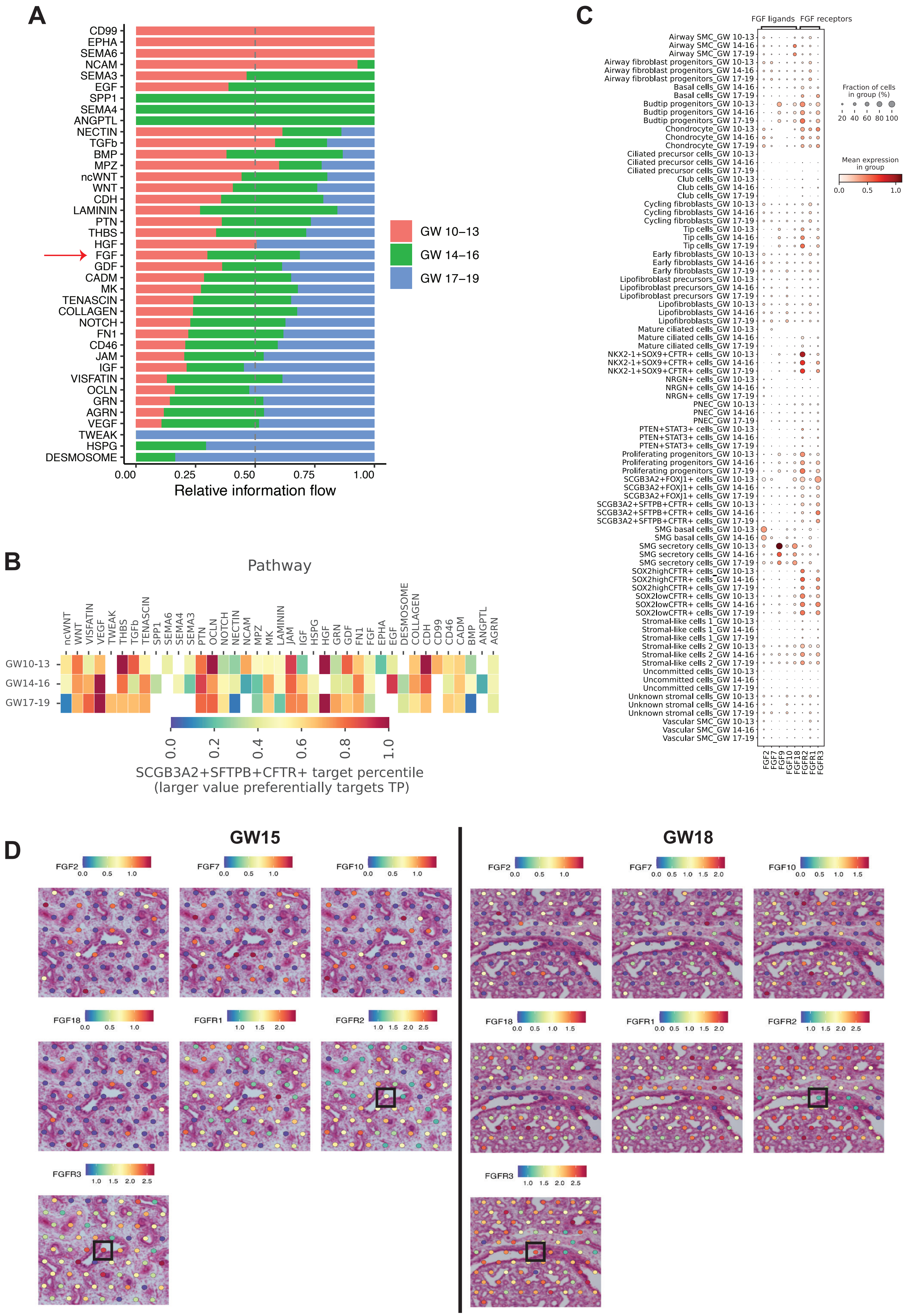

A
CD99
EPHA
SEMA6
NCAM
SEMA3
EGF
SPP1
SEMA4
ANGPTL
NECTIN
TGFb
BMP
MPZ
ncWNT
WNT
CDH
LAMININ
PTN
THBS
HGF
FGF
GDF
CADM
MK
TENASCIN
COLLAGEN
NOTCH
FN1
CD46
JAM
IGF
VISFATIN
OCLN
GRN
AGRN
VEGF
TWEAK
HSPG
DESMOSOME
0.00 0.25 0.50 0.75 1.00
Relative information flow
GW 10–13
GW 14–16
GW 17–19
B
Pathway
ncWNT WNT VISFATIN VEGF TWEAK THBS TGFb TENASCIN SPP1 SEMA6 SEMA4 SEMA3 PTN OCLN NOTCH NECTIN NCAM MPZ MK LAMININ JAM IGF HSPG HGF GRN GDF FN1 FGF EPHA EGF DESMOSOME COLLAGEN CDH CD99 CD46 CADM BMP ANGPTL AGRN
GW10-13
GW14-16
GW17-19
0.0 0.2 0.4 0.6 0.8 1.0
SCGB3A2+SFTPB+CFTR+ target percentile
(larger value preferentially targets TP)
C
FGF ligands FGF receptors
Airway SMC_GW 10-13
Airway SMC_GW 14-16
Airway SMC_GW 17-19
Airway fibroblast progenitors_GW 10-13
Airway fibroblast progenitors_GW 14-16
Airway fibroblast progenitors_GW 17-19
Basal cells_GW 14-16
Basal cells_GW 17-19
Budtip progenitors_GW 10-13
Budtip progenitors_GW 14-16
Budtip progenitors_GW 17-19
Chondrocyte_GW 10-13
Chondrocyte_GW 14-16
Chondrocyte_GW 17-19
Ciliated precursor cells_GW 10-13
Ciliated precursor cells_GW 14-16
Ciliated precursor cells_GW 17-19
Club cells_GW 10-13
Club cells_GW 14-16
Club cells_GW 17-19
Cycling fibroblasts_GW 10-13
Cycling fibroblasts_GW 14-16
Cycling fibroblasts_GW 17-19
Tip cells_GW 10-13
Tip cells_GW 14-16
Tip cells_GW 17-19
Early fibroblasts_GW 10-13
Early fibroblasts_GW 14-16
Early fibroblasts_GW 17-19
Lipofibroblast precursors_GW 10-13
Lipofibroblast precursors_GW 14-16
Lipofibroblast precursors_GW 17-19
Lipofibroblasts_GW 10-13
Lipofibroblasts_GW 14-16
Lipofibroblasts_GW 17-19
Mature ciliated cells_GW 10-13
Mature ciliated cells_GW 14-16
Mature ciliated cells_GW 17-19
NKX2-1+SOX9+CFTR+ cells_GW 10-13
NKX2-1+SOX9+CFTR+ cells_GW 14-16
NKX2-1+SOX9+CFTR+ cells_GW 17-19
NRGN+ cells_GW 10-13
NRGN+ cells_GW 14-16
NRGN+ cells_GW 17-19
PNEC_GW 10-13
PNEC_GW 14-16
PNEC_GW 17-19
PTEN+STAT3+ cells_GW 10-13
PTEN+STAT3+ cells_GW 14-16
PTEN+STAT3+ cells_GW 17-19
Proliferating progenitors_GW 10-13
Proliferating progenitors_GW 14-16
Proliferating progenitors_GW 17-19
SCGB3A2+FOXJ1+ cells_GW 10-13
SCGB3A2+FOXJ1+ cells_GW 14-16
SCGB3A2+FOXJ1+ cells_GW 17-19
SCGB3A2+SFTPB+CFTR+ cells_GW 10-13
SCGB3A2+SFTPB+CFTR+ cells_GW 14-16
SCGB3A2+SFTPB+CFTR+ cells_GW 17-19
SMG basal cells_GW 10-13
SMG basal cells_GW 14-16
SMG secretory cells_GW 10-13
SMG secretory cells_GW 14-16
SMG secretory cells_GW 17-19
SOX2highCFTR+ cells_GW 10-13
SOX2highCFTR+ cells_GW 14-16
SOX2highCFTR+ cells_GW 17-19
SOX2lowCFTR+ cells_GW 10-13
SOX2lowCFTR+ cells_GW 14-16
SOX2lowCFTR+ cells_GW 17-19
Stromal-like cells 1_GW 10-13
Stromal-like cells 1_GW 14-16
Stromal-like cells 1_GW 17-19
Stromal-like cells 2_GW 10-13
Stromal-like cells 2_GW 14-16
Stromal-like cells 2_GW 17-19
Uncommitted cells_GW 10-13
Uncommitted cells_GW 14-16
Uncommitted cells_GW 17-19
Unknown stromal cells_GW 10-13
Unknown stromal cells_GW 14-16
Unknown stromal cells_GW 17-19
Vascular SMC_GW 10-13
Vascular SMC_GW 14-16
Vascular SMC_GW 17-19
FGF2 FGF7 FGF9 FGF10 FGF18 FGFR2 FGFR1 FGFR3
Fraction of cells in group (%)
20 40 60 80 100
Mean expression in group
0.0 0.5 1.0
D
GW15
GW18
FGF2
FGF7
FGF10
FGF18
FGFR1
FGFR2
FGFR3

Supplementary Fig. 10: FGF cell signaling networks from senders to TP cells.

A: Relative information flow of signalling towards TP cells for early (GW10-13), mid (GW14-16), and late (GW17-19). Information flow is calculated as the sum of all cell interaction probabilities towards TP cells for neighboring cell types.

B: Overview of signaling pathways strength towards TP cells in early (GW 10-13), mid (GW 14-16), late (GW 17-19). Values show the signalling strength towards TP relative to other cell types (as a percentile), where a value of 1.0 indicates TP cells are the top receiver of the signalling pathway for the subset of neighboring cell types.

C. Expression of FGF ligands and receptors in specific epithelial and stromal subset of cells from scRNA-seq grouped by early (GW 10-13), mid (GW 14-16), and late (GW17-19) stages.

D. Expression of FGF ligands and receptors near TP cells (black box on H&E staining) using Visium spatial transcriptomics.

Source data are provided as a Source Data file.

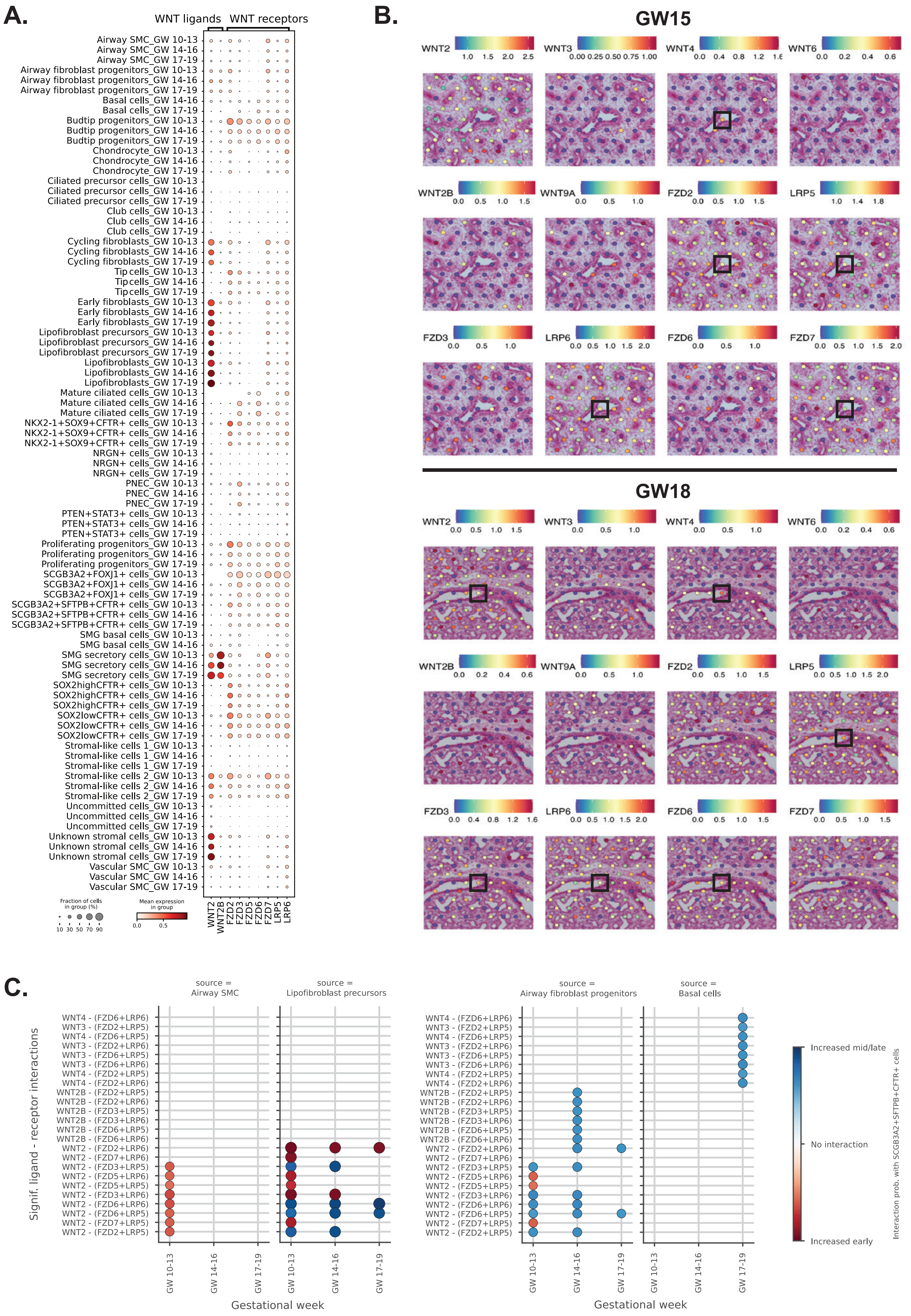
A.
WNT ligands
WNT receptors
Airway SMC_GW 10-13
Airway SMC_GW 14-16
Airway SMC_GW 17-19
Airway fibroblast progenitors_GW 10-13
Airway fibroblast progenitors_GW 14-16
Airway fibroblast progenitors_GW 17-19
Basal cells_GW 14-16
Basal cells_GW 17-19
Budtip progenitors_GW 10-13
Budtip progenitors_GW 14-16
Budtip progenitors_GW 17-19
Chondrocyte_GW 10-13
Chondrocyte_GW 14-16
Chondrocyte_GW 17-19
Ciliated precursor cells_GW 10-13
Ciliated precursor cells_GW 14-16
Ciliated precursor cells_GW 17-19
Club cells_GW 10-13
Club cells_GW 14-16
Club cells_GW 17-19
Cycling fibroblasts_GW 10-13
Cycling fibroblasts_GW 14-16
Cycling fibroblasts_GW 17-19
Tip cells_GW 10-13
Tip cells_GW 14-16
Tip cells_GW 17-19
Early fibroblasts_GW 10-13
Early fibroblasts_GW 14-16
Early fibroblasts_GW 17-19
Lipofibroblast precursors_GW 10-13
Lipofibroblast precursors_GW 14-16
Lipofibroblast precursors_GW 17-19
Lipofibroblasts_GW 10-13
Lipofibroblasts_GW 14-16
Lipofibroblasts_GW 17-19
Mature ciliated cells_GW 10-13
Mature ciliated cells_GW 14-16
Mature ciliated cells_GW 17-19
NKX2-1+SOX9+CFTR+ cells_GW 10-13
NKX2-1+SOX9+CFTR+ cells_GW 14-16
NKX2-1+SOX9+CFTR+ cells_GW 17-19
NRGN+ cells_GW 10-13
NRGN+ cells_GW 14-16
NRGN+ cells_GW 17-19
PNEC_GW 10-13
PNEC_GW 14-16
PNEC_GW 17-19
PTEN+STAT3+ cells_GW 10-13
PTEN+STAT3+ cells_GW 14-16
PTEN+STAT3+ cells_GW 17-19
Proliferating progenitors_GW 10-13
Proliferating progenitors_GW 14-16
Proliferating progenitors_GW 17-19
SCGB3A2+FOXJ1+ cells_GW 10-13
SCGB3A2+FOXJ1+ cells_GW 14-16
SCGB3A2+FOXJ1+ cells_GW 17-19
SCGB3A2+SFTPB+CFTR+ cells_GW 10-13
SCGB3A2+SFTPB+CFTR+ cells_GW 14-16
SCGB3A2+SFTPB+CFTR+ cells_GW 17-19
SMG basal cells_GW 10-13
SMG basal cells_GW 14-16
SMG secretory cells_GW 10-13
SMG secretory cells_GW 14-16
SMG secretory cells_GW 17-19
SOX2highCFTR+ cells_GW 10-13
SOX2highCFTR+ cells_GW 14-16
SOX2highCFTR+ cells_GW 17-19
SOX2lowCFTR+ cells_GW 10-13
SOX2lowCFTR+ cells_GW 14-16
SOX2lowCFTR+ cells_GW 17-19
Stromal-like cells 1_GW 10-13
Stromal-like cells 1_GW 14-16
Stromal-like cells 1_GW 17-19
Stromal-like cells 2_GW 10-13
Stromal-like cells 2_GW 14-16
Stromal-like cells 2_GW 17-19
Uncommitted cells_GW 10-13
Uncommitted cells_GW 14-16
Uncommitted cells_GW 17-19
Unknown stromal cells_GW 10-13
Unknown stromal cells_GW 14-16
Unknown stromal cells_GW 17-19
Vascular SMC_GW 10-13
Vascular SMC_GW 14-16
Vascular SMC_GW 17-19
Fraction of cells in group (%)
10 30 50 70 90
Mean expression in group
0.0 0.5
WNT2 WNT2B FZD2 FZD3 FZD5 FZD6 FZD7 LRP5 LRP6
B.
GW15
WNT2 1.0 1.5 2.0 2.5
WNT3 0.00 0.25 0.50 0.75 1.00
WNT4 0.0 0.4 0.8 1.2 1.6
WNT6 0.0 0.2 0.4 0.6
WNT2B 0.0 0.5 1.0 1.5
WNT9A 0.0 0.5 1.0
FZD2 0.0 0.5 1.0 1.5
LRP5 1.0 1.4 1.8
FZD3 0.0 0.5 1.0
LRP6 0.0 0.5 1.0 1.5 2.0
FZD6 0.0 0.5 1.0
FZD7 0.0 0.5 1.0 1.5 2.0
GW18
WNT2 0.0 0.5 1.0 1.5
WNT3 0.0 0.5 1.0
WNT4 0.0 0.5 1.0
WNT6 0.0 0.2 0.4 0.6
WNT2B 0.0 0.2 0.4 0.6
WNT9A 0.0 0.5 1.0
FZD2 0.0 0.5 1.0 1.5
LRP5 0.0 0.5 1.0 1.5 2.0
FZD3 0.0 0.4 0.8 1.2 1.6
LRP6 0.0 0.5 1.0 1.5 2.0
FZD6 0.0 0.5 1.0 1.5
FZD7 0.0 0.5 1.0 1.5
C.
source = Airway SMC
source = Lipofibroblast precursors
source = Airway fibroblast progenitors
source = Basal cells
Signif. ligand - receptor interactions
WNT4 - (FZD6+LRP6)
WNT3 - (FZD2+LRP5)
WNT4 - (FZD6+LRP5)
WNT3 - (FZD2+LRP6)
WNT3 - (FZD6+LRP5)
WNT3 - (FZD6+LRP6)
WNT4 - (FZD2+LRP5)
WNT4 - (FZD2+LRP6)
WNT2B - (FZD2+LRP5)
WNT2B - (FZD2+LRP6)
WNT2B - (FZD3+LRP5)
WNT2B - (FZD3+LRP6)
WNT2B - (FZD6+LRP5)
WNT2B - (FZD6+LRP6)
WNT2 - (FZD2+LRP6)
WNT2 - (FZD7+LRP6)
WNT2 - (FZD3+LRP5)
WNT2 - (FZD5+LRP6)
WNT2 - (FZD5+LRP5)
WNT2 - (FZD3+LRP6)
WNT2 - (FZD6+LRP6)
WNT2 - (FZD6+LRP5)
WNT2 - (FZD7+LRP5)
WNT2 - (FZD2+LRP5)
GW 10-13
GW 14-16
GW 17-19
Gestational week
Increased mid/late
No interaction
Increased early
Interaction prob. with SCGB3A2+SFTPB+CFTR+ cells

Supplementary Fig. 11: WNT signalling from senders to TP cells.

A: Expression of WNT ligands and receptors in specific epithelial and stromal subset of cells from scRNA-seq grouped by early (GW 10-13), mid (GW 14-16), and late (GW17-19) stages.

B: Expression of WNT ligands and receptors near TP cells (black box on H&E staining) using Visium spatial transcriptomics.

C: Ligand-receptor (L-R) plot showcasing specific WNT ligand-receptor interactions enriched in early vs mid/late from airway SMC, lipofibroblast precursors, airway fibroblast progenitors, and basal cells to TP cells. Red values show significantly increased early; blue values show interactions significantly increased mid/late. Significance is determined using a permutation test randomly permuting cell type labels, significant interactions are chosen with $p < 0.01$.

Source data are provided as a Source Data file.

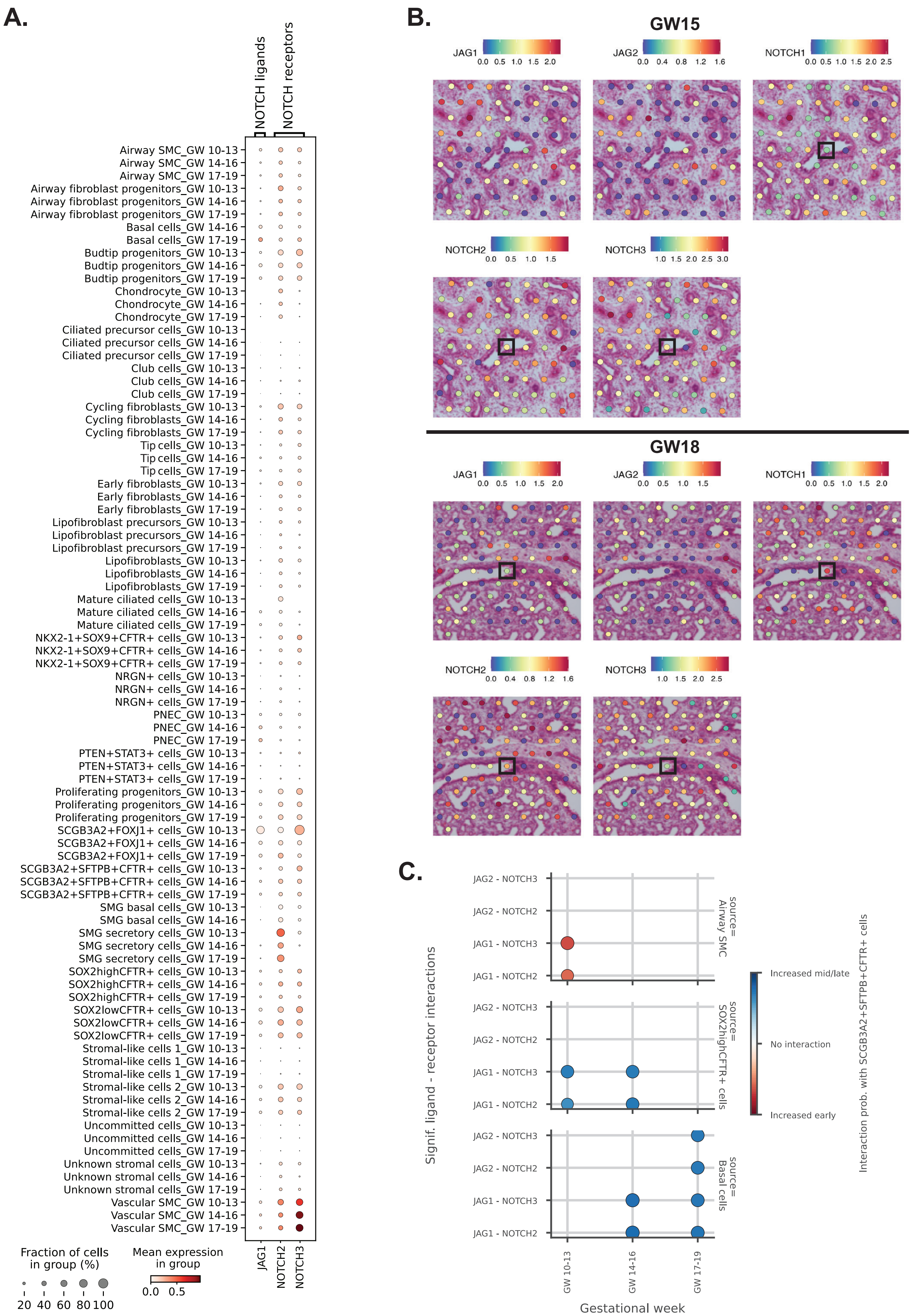
A.
NOTCH ligands
NOTCH receptors
Airway SMC_GW 10-13
Airway SMC_GW 14-16
Airway SMC_GW 17-19
Airway fibroblast progenitors_GW 10-13
Airway fibroblast progenitors_GW 14-16
Airway fibroblast progenitors_GW 17-19
Basal cells_GW 14-16
Basal cells_GW 17-19
Budtip progenitors_GW 10-13
Budtip progenitors_GW 14-16
Budtip progenitors_GW 17-19
Chondrocyte_GW 10-13
Chondrocyte_GW 14-16
Chondrocyte_GW 17-19
Ciliated precursor cells_GW 10-13
Ciliated precursor cells_GW 14-16
Ciliated precursor cells_GW 17-19
Club cells_GW 10-13
Club cells_GW 14-16
Club cells_GW 17-19
Cycling fibroblasts_GW 10-13
Cycling fibroblasts_GW 14-16
Cycling fibroblasts_GW 17-19
Tip cells_GW 10-13
Tip cells_GW 14-16
Tip cells_GW 17-19
Early fibroblasts_GW 10-13
Early fibroblasts_GW 14-16
Early fibroblasts_GW 17-19
Lipofibroblast precursors_GW 10-13
Lipofibroblast precursors_GW 14-16
Lipofibroblast precursors_GW 17-19
Lipofibroblasts_GW 10-13
Lipofibroblasts_GW 14-16
Lipofibroblasts_GW 17-19
Mature ciliated cells_GW 10-13
Mature ciliated cells_GW 14-16
Mature ciliated cells_GW 17-19
NKX2-1+SOX9+CFTR+ cells_GW 10-13
NKX2-1+SOX9+CFTR+ cells_GW 14-16
NKX2-1+SOX9+CFTR+ cells_GW 17-19
NRGN+ cells_GW 10-13
NRGN+ cells_GW 14-16
NRGN+ cells_GW 17-19
PNEC_GW 10-13
PNEC_GW 14-16
PNEC_GW 17-19
PTEN+STAT3+ cells_GW 10-13
PTEN+STAT3+ cells_GW 14-16
PTEN+STAT3+ cells_GW 17-19
Proliferating progenitors_GW 10-13
Proliferating progenitors_GW 14-16
Proliferating progenitors_GW 17-19
SCGB3A2+FOXJ1+ cells_GW 10-13
SCGB3A2+FOXJ1+ cells_GW 14-16
SCGB3A2+FOXJ1+ cells_GW 17-19
SCGB3A2+SFTPB+CFTR+ cells_GW 10-13
SCGB3A2+SFTPB+CFTR+ cells_GW 14-16
SCGB3A2+SFTPB+CFTR+ cells_GW 17-19
SMG basal cells_GW 10-13
SMG basal cells_GW 14-16
SMG secretory cells_GW 10-13
SMG secretory cells_GW 14-16
SMG secretory cells_GW 17-19
SOX2highCFTR+ cells_GW 10-13
SOX2highCFTR+ cells_GW 14-16
SOX2highCFTR+ cells_GW 17-19
SOX2lowCFTR+ cells_GW 10-13
SOX2lowCFTR+ cells_GW 14-16
SOX2lowCFTR+ cells_GW 17-19
Stromal-like cells 1_GW 10-13
Stromal-like cells 1_GW 14-16
Stromal-like cells 1_GW 17-19
Stromal-like cells 2_GW 10-13
Stromal-like cells 2_GW 14-16
Stromal-like cells 2_GW 17-19
Uncommitted cells_GW 10-13
Uncommitted cells_GW 14-16
Uncommitted cells_GW 17-19
Unknown stromal cells_GW 10-13
Unknown stromal cells_GW 14-16
Unknown stromal cells_GW 17-19
Vascular SMC_GW 10-13
Vascular SMC_GW 14-16
Vascular SMC_GW 17-19
JAG1
NOTCH2
NOTCH3
Fraction of cells in group (%)
20 40 60 80 100
Mean expression in group
0.0 0.5
B.
GW15
JAG1
0.0 0.5 1.0 1.5 2.0
JAG2
0.0 0.4 0.8 1.2 1.6
NOTCH1
0.0 0.5 1.0 1.5 2.0 2.5
NOTCH2
0.0 0.5 1.0 1.5
NOTCH3
1.0 1.5 2.0 2.5 3.0
GW18
JAG1
0.0 0.5 1.0 1.5 2.0
JAG2
0.0 0.5 1.0 1.5
NOTCH1
0.0 0.5 1.0 1.5 2.0
NOTCH2
0.0 0.4 0.8 1.2 1.6
NOTCH3
1.0 1.5 2.0 2.5
C.
JAG2 - NOTCH3
JAG2 - NOTCH2
JAG1 - NOTCH3
JAG1 - NOTCH2
source= Airway SMC
source= SOX2highCFTR+ cells
source= Basal cells
Signif. ligand - receptor interactions
GW 10-13
GW 14-16
GW 17-19
Gestational week
Increased mid/late
No interaction
Increased early
Interaction prob. with SCGB3A2+SFTPB+CFTR+ cells

Supplementary Fig. 12: NOTCH signalling from senders to TP cells.

A: Expression of NOTCH ligands and receptors in specific epithelial and stromal subset of cells from scRNA-seq grouped by early (GW 10-13), mid (GW 14-16), and late (GW17-19) stages.

B: Expression of NOTCH ligands and receptors near TP cells (black box on H&E staining) using Visium spatial transcriptomics.

C: Ligand-receptor (L-R) plot showcasing specific NOTCH ligand-receptor interactions enriched in early vs mid/late from airway SMC, $SOX2^{high}CFTR+$, and basal cells to TP cells. Red values show interactions significantly increased early; blue values show interactions significantly increased mid/late. Significance is determined using a permutation test randomly permuting cell type labels, significant interactions are chosen with $p < 0.01$.

Source data are provided as a Source Data file.

Supplementary Notes

Supplementary Note 1. Comparison to recently published fetal lung datasets.

We performed the MapQuery[1] function to tease out the specific cellular similarities identified in our dataset compared to each of the recently published fetal lung cell datasets by He et al.[2], Sountoulidis et al.[3], and Cao et al[4]. By setting the previous fetal lung datasets as the reference, we visualized transcriptomic similarity through plotting our cells onto the reference dataset's UMAP and calculated the percentage of cell cluster overlap to be 105/144 (73%), 64/75 (85%), and 31/33 (94%) for He et al.[2], Sountoulidis et al.[3], and Cao et al[4] datasets, respectively (Supplementary Fig. 2A, B, C). Cell types not found in our dataset include cells predominantly in earlier gestational fetal lung tissues that we did not collect (<GW10), and include cell such as early tip and early stalk cells by He et al. In addition, cells predominantly found in the large airways including the cartilaginous tissues (i.e., tracheal regions) were not captured in our dataset (i.e., prox. progenitor 1 and prox. secretory from Sountoulidis et al.).

Supplementary Note 2. Co-development of the fetal pulmonary endothelium.

The fetal pulmonary endothelium contained 10 endothelial subtypes in the early developing lung (Supplementary Fig. 3A) that expressed pan-endothelial gene *PECAM1*. These endothelial cell (EC) subtypes included erythrocyte-like EC-1, *KIT+CA4+* EC, erythrocyte-like EC-2, intermediate lymphatic EC, lymphatic EC, venous EC, aerocytes, general capillaries (gCap), arterial EC, and cycling early capillaries. Both EC-1/EC-2 expressed high levels of hemoglobin genes and were annotated as erythrocyte-like cell populations (Supplementary Data 1). The proportion of each EC subtypes remained relatively consistent in all the gestational timepoint studied (Supplementary Fig. 3B). Notably, the fetal lung lymphatic cells were found as early as GW10 and continued to develop with time, which would become important in neonatal lung respiration[5]. The top DEG expressed in both lymphatic populations included *PROX1*, *CCL21* and *TFF3*[6,7], with the lymphatic EC expressing higher levels of genes associated with inflammation *PTX3* and *NRP2* (Supplementary Fig. 3 and Supplementary Data 1). Differential enrichment analysis of the transcription factors driving these cell lineages was performed using SCENIC[8] (Supplementary Fig. 3D). Cycling early capillaries expressed high levels of genes associated with cellular proliferation such as *TOP2A, MKI67*, and *CENPF.* Transcription factors regulating cell proliferation and cell cycle *BRCA1, E2F7, MYBL2* were highly active in the cycling early capillaries. Aerocytes and gCap cells shared similar DEG with gCap expressing higher levels of *HBEGF,* previously shown to promote angiogenesis[9], while aerocytes differentially expressed high levels of *EGFL6* and *COL6A3*. Interestingly, aerocytes shared similar differentially expressed transcription factors as gCap, including *REL, IRF, POU2F2,* suggesting these two cell types may share developmental origins, as in adult mouse lungs[10]. Higher expression of *HGPD, CA4,* and *KIT* distinguished the *KIT+CA4+* cells from the EC-1 and EC-2 cells. Higher regulon activity of the transcription factors *MLX, SOX18* and *ZNF71* were also observed in *KIT+CA4+* EC. Venous EC expressed high levels of the specific pulmonary venous gene *CPE*[7] and the apelin receptor, *APLNR*, the latter regulates endothelial cell differentiation[11]. Arterial EC expressed abundant *DKK2, CXCL12* and the transcription factors *CREB1* and *SMAD4,* the latter two important in the formation and function of the vasculature[12,13].

To determine the developmental relationships between these cells, we used our RNA velocity-based method LatentVelo[14] (Supplementary Fig. 3 and E', respectively) and visualized velocities using partition-based graph abstraction (PAGA) of RNA velocity projections (Supplementary Fig. 3F). In brief, velocities infer the directionality of lineage development and corresponding latent times identify the position of cells along the developmental trajectory, shown as a dark blue to yellow hue (Supplementary Fig. 3E'). In doing so, cell origins were determined, and trajectories predicted based on these relationships over time. Several conserved trajectories were found, but also unique trajectories stemming from putative multipotent EC. Focusing on capillary specification, we identified a lineage relationship between cycling early capillaries to gCap cells and aerocytes with a gradual change in top DEGs associated with these cell lineages as they differentiate. We found an increase in CXCL12, and EDNRB associated with gCap and aerocyte differentiation respectively (Supplementary Fig. 3G). Xenium spatial transcriptomics showed localization of CA4+STC1+ cells and THY1+ cells associated with gCap and aerocytes in KDR+ endothelial cells, respectively (Supplementary Fig. 3H). gCap cells were found closely associated with the airway epithelium, while aerocytes occupied its own space. The lineage development of gCap and aerocytes has also been found in murine neonatal alveolar lung[10].

Supplementary Note 3. Novel alveolar macrophage lineage trajectories identified in the fetal lung.

The fetal lung immune population contained 18 distinct cell types (Supplementary Fig. 4A). These included a common lymphoid progenitor, proliferating macrophage/monocytes, monocyte 1 and 2, alveolar macrophage 2 (aM2), aM1, aM-like cell, B cells, cycling B cells, plasma cells, monocyte/macrophage precursor cells, *CD1C+* dendritic cells 1 (DC), macrophage, *FLT3+* preDC, *IL7R+* T cells, immature double positive (DP) leukocytes, cycling lymphocytes, and plasmacytoid DC (pDC). The proportion of mature cell types including monocytes, plasma cells and B cells increased in later gestational lung tissues (Supplementary Fig. 4B). Top genes differentially expressed in each subtype distinctly separates the different clusters (Supplementary Fig. 4C and Supplementary Data 1). Gene ontology enrichment analysis of the DEG (Supplementary Fig. 4D) showed terms associated with antigen processing and presentation and major histocompatibility complex (MHC) class II protein in all DC lineages. Dendritic cells made up a smaller proportion of myeloid cells in the fetal lungs. Their role in immunity during development is unknown. However, studies have suggested fetal DC may help induce immunologic tolerance where fetal and maternal immune cells may come into contact during pregnancy[15]. *CD1C+* DC and *FLT3+* DC share several common genes mainly from the HLA family. *CD1C+* DC have previously been shown to prime cytotoxic T cell responses[16], while *FLT3+* DC may mark an early developing DC population as the FLT3 ligand has previously been shown to regulate dendritic cell development[17]. Transcription factors differentially enriched (Supplementary Fig. 4E) in each cell cluster identified unique expression of acetyl-CoA carboxylase 1 (*ACAA1)* in *CD1C+* DC and Basic Leucine Zipper ATF-Like Transcription Factor 3 *(BATF3)* in *FLT3+* DC, both previously shown to regulate DC development and function[18,19], respectively. Spatial transcriptomic showed both monocytes (*FCN1*, *CSTA*) and macrophages (*CD86, and CD68,* presumably alveolar macrophages) sparsely distributed in both GW 15 and 18 fetal lung tissue (Supplementary Fig. 4F).

The AT-rich interaction domain 3A *(ARID3A)* transcription factor was differentially enriched in pDC, as previously observed[20]. Plasmacytoid DC are rare cells that secrete large

amounts of type I interferons with a role in antiviral immunity and share similar morphology as plasma cells[21]. They also play a role in inducing T cell tolerance and therefore may exist in the developing lung to suppress alloreactive T cells. It is unclear if these pDC originated in the developing lungs or as previous studies have shown, are bone marrow-derived and circulated into the lungs[22]. Nonetheless, pDC play an important role in immune responses to inhaled antigens[23]. LatentVelo identified a lineage trajectory originating from pDC to *CD1C*+ DC to aM2 cells and aM1 cells, and a trajectory from *FLT3*+ preDC to *CD1C*+ DC (Supplementary Fig. 4G-H). GO terms associated with inflammatory and immune responses were enriched in aM1, aM2 and AM-like clusters (Supplementary Fig. 4D). Both aM1/aM2 are delineated based on *CD68* expression, a classical marker of monocyte-derived alveolar macrophages, and were distinguished between one another based on expression of proinflammatory genes *IL1B, TNF* and anti-inflammatory *IL10* and *TGFB* genes. On the contrary, aM-like cells expressed *IL1B* but shared no other proinflammatory genes as aM1. Alveolar macrophages (aM-like, aM1, aM2) shared similar elevated expression of *POU2F2* and *NFKB1* suggesting a common conserved developmental regulation (Supplementary Fig. 4E). Regulation of *NFKB1* signalling in fetal macrophages is important for airway branching as it protects from LPS-induced proinflammatory responses[24,25]. High expression of *SMAD6* distinguished aM-like from aM1 and aM2 cells, while *KLF11* expression was higher in aM2 cells. Based on Xenium spatial analysis, *CD68, CD86, FCN1,* and *CSTA* were sparsely localized in the surrounding stroma (Supplementary Fig. 4F). LatentVelo and Slingshot analyses (Supplementary Fig. 4G, G') showed a lineage trajectory originating from monocyte/macrophage precursor to aM-like cells to aM2 cells and aM1 cells. This is confirmed with a corresponding change in top DEG as the cells differentiate along the trajectories (Supplementary Fig. 4H and I).

B cells, cycling B cells, and plasma cells express the canonical B cell signalling molecules CD79B, VREB3[26] and GO terms associated with B cell receptor signaling pathway and B cell activation (Supplementary Fig. 4D). Indeed, bacterial LPS is a potent stimulant of B cell and macrophage differentiation and antibody secretion[27]. LatentVelo and Slingshot analyses identified a lineage trajectory originating from B cells to plasma cells, and trajectories from monocyte/macrophage precursor to monocyte 1 and monocyte 2 (Supplementary Fig. 4I).

Supplementary Note 4. Additional analyses of the temporal changes in NOTCH and WNT signalling in the TP cells.

WNT signaling in TP cells were found to be elevated in early (GW 10-13) stages and decreased in mid and late stages (10B). WNT receptors *FZD2, FZD3, FZD5, FZD6, FZD7, LRP5, LRP6* were found to be expressed in TP cells with comparatively higher expression of *FZD2* and *FZD7* in the early stages (Supplementary Fig. 11A). Based on spatial expression from Visium, we identified *FZD2, LRP5, LRP6,* and *FZD7* expressed in areas of abundant TP spots at GW15, whereas *LRP5, FZD3, LRP6,* and *FZD6* were expressed at GW18 (Supplementary Fig. 11B). Sources of WNT signalling was assessed in early, mid, late stages to TP cells where *WNT2* was expressed in multiple cell types including airway SMC (only in early stage), lipofibroblast precursors, airway fibroblast progenitors, and basal cells (Supplementary Fig. 11C). Signals from basal cells appeared to dominate in late tissues, although basal cell spatial proximity to TP cells decreases in these late tissues (Fig. 7E), so we cannot be confident in this interaction.

We also assessed NOTCH signaling in TP cells and found relatively strong NOTCH signaling in lung tissues during the mid (GW14-16) and late (GW17-19) timepoints (Supplementary Fig. 10B). NOTCH receptors, *NOTCH2* and *NOTCH3,* were expressed in multiple cell types including TP cells and *SCGB3A2+FOXJ1+* cells, but not in PNEC (Supplementary Fig. 12A). NOTCH ligand *JAG1* was expressed in multiple cell types including vascular SMC, *SOX2*$^{high/low}$*CFTR+* cells, and basal cells. High expression of *NOTCH2* and *NOTCH3* was found in spots with high proportions of TP cells (black box, Supplementary Fig. 12B). Several sources of NOTCH signaling with TP cells included airway SMC, *SOX2*^{high}*CFTR+* cells, and basal cells (Supplementary Fig. 12C). Airway SMC was a source of NOTCH signaling during the early stage that was lost in mid/late stages. Increased *JAG1-NOTCH2* and *JAG1-NOTCH3* L-R interactions are also observed in mid stage tissues stemming from *SOX2*^{high}*CFTR+* cells. NOTCH signalling also increased in late stage tissues due to signaling from basal cells, however basal cells lose their proximity to TP cells in later tissues (Fig. 7E). Overall, our data suggests NOTCH-mediated signaling to TP during mid stage may involve direct interaction with *SOX2*^{high}*CFTR+* cells.

Supplementary Note 5. Discussion on CFTR expressing cells

The pseudoglandular lung is marked by extensive branching morphogenesis, where the cells must undergo extensive proliferation, proximal-distal patterning, cell migration, and differentiation into specialized cell types. A major finding in our analysis is the identification of several progenitor cells expressing high levels of *CFTR*. These cells include the *NKX2-1+SOX9+CFTR+, SOX2*^{high}*CFTR+, SOX2*^{low}*CFTR+,* and TP cells. The co-expression of *CFTR* in these progenitor cell types suggest a putative role for CFTR in branching morphogenesis and the formation of specific epithelial cell lineages. In the postnatal lung, mutations in *CFTR* can cause cystic fibrosis (CF), a disease that manifests in the airways and impairs lung function and ultimate destruction. The *CFTR* gene encodes for a chloride channel that regulates water and ion transport across the epithelium. The precise role of CFTR in the developing lung is unclear but the early expression of *CFTR* may suggest a role for this protein, and the cells that expresses it, in airway formation. Moreover, understanding the role of CFTR in airway cell development may provide important insight into the early manifestations of CF lung pathogenesis and the long-term impact on disease progression.

Supplementary Tables

Supplementary Table 1: Detailed list of fetal lung tissue samples sequenced.

Sample ID (Gestational week_+ day)	Sex	# of sequenced cells	# of UMIs	Filtered # of cells	% change of cells
GW_10_1	Female	13852	27559	12412	0.103956107
GW_10_2	Male	5937	17271	5515	0.07107967
GW_11_1	Male	4923	16935	4629	0.059719683
GW_12_3	Male	10689	17067	10277	0.038544298
GW_13_4	Male	3748	16573	3588	0.042689434
GW_13_6	Male	13464	17276	13054	0.030451575
GW_14_3	Male	3916	16255	3748	0.042900919
GW_15_5_1	Female	15444	18067	8168	0.471121471
GW_15_5_2	Female	18849	17214	13347	-0.508306023
GW_16_1	Male	6425	16835	5636	0.122801556
GW_16_2	Female	7687	16415	7380	0.039937557
GW_16_5	Female	5524	24481	4566	0.173425054
GW_18	Male	10505	16700	10477	0.002665397
GW_18_1	Male	12109	16855	10876	0.101825089
GW_18_2	Female	6330	16223	6060	0.042654028
*GW_18_1A	Male	7621	17182	6920	0.091982679
*GW_18_1B	Male	6341	17061	5888	0.071439836
GW_19_0	Male	10565	17664	9764	0.075816375
GW_19_2	Female	6847	16970	6107	0.10807653
GW_19_4	Male	9480	25027	8286	0.125949367
Total count	7 Female<br>12 Male	170256		156698	

*Same gestational lung tissue. "A" and "B" indicates different regions (lower and upper regions) of the lung tissue sampled for sequencing.

Supplementary Table 2: List of shared and unique cell types compared to published datasets: He et al., Sountoulidis et al., and Cao et al.

MapQuery results of publicly available and Quach et al. lung data set	
Cell identity	Number of queried cells
Aerocyte	14
Airway fibroblast progenitors	22441
Airway SMC	24286
aM-like cells	67
aM1 cells	598
aM2 cells	1038
Arterial EC	921
B-cells	208
Budtip progenitors	2164
CD1C+ DC	1340
Chondrocyte	755
Ciliated precursor cells	232
Club cells	74
Common lymphoid progenitor (CLP)	2766
Cycling B cells	2454
Cycling early capillaries	2355
Cycling fibroblasts	43955
Cycling lymphocytes	883
Tip cells	7968
Early fibroblasts	50296
Erythrocyte-like EC-1	537
Erythrocyte-like EC-2	3963
FLT3+ preDC	247
gCap	416
IL7R+ T cells	1209
Immature DP leukocytes	302
Intermediate lymphatic EC	1237
KIT+CA4+ endothelial cells	1076
Lipofibroblast precursors	38500
Lipofibroblasts	1542
Lymphatic EC	251
Macrophage	891
Monocyte 1	771
Monocyte/macrophage precursor cells	2160
NKX2-1+SOX9+CFTR+ cells	1197
NRGN+ cells	215
pDC	261
Plasma cell	1493

PNEC	2173
Proliferating macrophage/monocyte cells	3254
Proliferating progenitors	10305
PTEN+STAT3+ cells	160
SCGB3A2+FOXJ1+ cells	1540
SCGB3A2+SFTPB+CFTR+ cells	2190
Schwann	5371
SMG secretory cells	567
SOX2highCFTR+ cells	351
SOX2lowCFTR+ cells	3606
Stromal-like cells 1	23
Stromal-like cells 2	4
Uncommitted cells	706
Unknown stromal cells	2312
Vascular SMC	3466
Venous EC	875
Undetected cells	
Basal cells	
Mature ciliated cells	
Monocyte 2	
SMG basal cells	

Supplementary Table 3: List of primary and secondary antibodies.

Primary Antibodies	Host	Supplier	Cat. no.	Dilution
ACTA2	Rabbit	Abcam	ab124964	1:500
ASCL1	Goat	Rockland	600-101-MH4	1:500
CFTR	Mouse	Millipore	MAB1660	1:70
Delta NP63	Mouse	Abcam	ab735	1:100
Desmin	Goat	R&D	AF3844-SP	1:100
FOXJ1	Rabbit	Abcam	ab235445	1:100
MEF2C	Mouse	Invitrogen	MA5-25477	1:50
NKX2-1	Rabbit	Abcam	ab76013	1:200
SCGB3A2	Goat	Santa Cruz	sc-48320	1:50
SFTPB	Rabbit	Abcam	ab40876	1:50
SOX2	Goat	R&D	AF2018	1:300
SOX9	Goat	R&D	AF3075	1:600

Secondary Antibodies	Host	Supplier	Cat. no.	Dilution
Anti-mouse IgG (H+L) Alexa Fluor 488	Donkey	Invitrogen	A21202	1:500
Anti-rabbit IgG (H+L) Alexa Fluor 488	Donkey	Invitrogen	A21206	1:500
Anti-rat IgG (H+L) Alexa Fluor 488	Donkey	Invitrogen	A21208	1:500
Anti-mouse IgG (H+L) Alexa Fluor 546	Donkey	Invitrogen	A10036	1:500
Anti-rabbit IgG (H+L) Alexa Fluor 546	Donkey	Invitrogen	A10040	1:500
Anti-goat IgG (H+L) Alexa Fluor 546	Donkey	Invitrogen	A11056	1:500
Anti-rat IgG (H+L) Alexa Fluor 555	Donkey	Invitrogen	A48270	1:500
Anti-Rabbit IgG (H&L) Alexa Fluor 647	Donkey	Abcam	ab150075	1:500
Anti-goat IgG (H+L) Alexa Fluor 647	Donkey	Invitrogen	A21447	1:500

Supplementary References

1. Hao, Y. *et al.* Integrated analysis of multimodal single-cell data. *Cell* **184**, 3573-3587.e29 (2021).

2. He, P. *et al.* A human fetal lung cell atlas uncovers proximal-distal gradients of differentiation and key regulators of epithelial fates. *Cell* **185**, 4841-4860.e25 (2022).

3. Sountoulidis, A. *et al.* A topographic atlas defines developmental origins of cell heterogeneity in the human embryonic lung. *Nat Cell Biol* **25**, 351–365 (2023).

4. Cao, S. *et al.* Single-cell RNA sequencing reveals the developmental program underlying proximal–distal patterning of the human lung at the embryonic stage. *Cell Res* **33**, 421–433 (2023).

5. Jakus, Z. *et al.* Lymphatic function is required prenatally for lung inflation at birth. *J Exp Med* **211**, 815–826 (2014).

6. Takeda, A. *et al.* Single-Cell Survey of Human Lymphatics Unveils Marked Endothelial Cell Heterogeneity and Mechanisms of Homing for Neutrophils. *Immunity* **51**, 561-572.e5 (2019).

7. Schupp, J. C. *et al.* Integrated Single-Cell Atlas of Endothelial Cells of the Human Lung. *Circulation* **144**, 286–302 (2021).

8. Aibar, S. *et al.* SCENIC: single-cell regulatory network inference and clustering. *Nat Methods* **14**, 1083–1086 (2017).

9. Mehta, V. B. & Besner, G. E. HB-EGF promotes angiogenesis in endothelial cells via PI3-kinase and MAPK signaling pathways. *Growth Factors* **25**, 253–263 (2007).

10. Gillich, A. *et al.* Capillary cell-type specialization in the alveolus. *Nature* **586**, 785–789 (2020).

11. Masoud, A. G. *et al.* Apelin directs endothelial cell differentiation and vascular repair following immune-mediated injury. *J Clin Invest* **130**, 94–107 (2020).

12. Poduri, A. *et al.* Endothelial cells respond to the direction of mechanical stimuli through SMAD signaling to regulate coronary artery size. *Development* **144**, 3241–3252 (2017).

13. Li, L. *et al.* The α and Δ isoforms of CREB1 are required to maintain normal pulmonary vascular resistance. *PLoS One* **8**, e80637 (2013).

14. Farrell, S., Mani, M. & Goyal, S. Inferring single-cell transcriptomic dynamics with structured latent gene expression dynamics. 2022.08.22.504858 Preprint at https://doi.org/10.1101/2022.08.22.504858 (2022).

15. Blois, S. M. *et al.* Dendritic cells: key to fetal tolerance? *Biol Reprod* **77**, 590–598 (2007).

16. Nizzoli, G. *et al.* Human CD1c+ dendritic cells secrete high levels of IL-12 and potently prime cytotoxic T-cell responses. *Blood* **122**, 932–942 (2013).

17. Karsunky, H., Merad, M., Cozzio, A., Weissman, I. L. & Manz, M. G. Flt3 ligand regulates dendritic cell development from Flt3+ lymphoid and myeloid-committed progenitors to Flt3+ dendritic cells in vivo. *J Exp Med* **198**, 305–313 (2003).

18. Spranger, S., Dai, D., Horton, B. & Gajewski, T. F. Tumor-Residing Batf3 Dendritic Cells Are Required for Effector T Cell Trafficking and Adoptive T Cell Therapy. *Cancer Cell* **31**, 711-723.e4 (2017).

19. Nguyen-Phuong, T., Chung, H., Jang, J., Kim, J.-S. & Park, C.-G. Acetyl-CoA carboxylase-1/2 blockade locks dendritic cells in the semimature state associated with FA deprivation by favoring FAO. *J Leukoc Biol* **111**, 539–551 (2022).

20. Ratliff, M. L. *et al.* ARID3a gene profiles are strongly associated with human interferon alpha production. *J Autoimmun* **96**, 158–167 (2019).

21. Lee, Y. S. & Radford, K. J. The role of dendritic cells in cancer. *Int Rev Cell Mol Biol* **348**, 123–178 (2019).

22. Zuniga, E. I., McGavern, D. B., Pruneda-Paz, J. L., Teng, C. & Oldstone, M. B. A. Bone marrow plasmacytoid dendritic cells can differentiate into myeloid dendritic cells upon virus infection. *Nat Immunol* **5**, 1227–1234 (2004).

23. de Heer, H. J. *et al.* Essential role of lung plasmacytoid dendritic cells in preventing asthmatic reactions to harmless inhaled antigen. *J Exp Med* **200**, 89–98 (2004).

24. Blackwell, T. S. *et al.* NF-κB signaling in fetal lung macrophages disrupts airway morphogenesis. *J Immunol* **187**, 2740–2747 (2011).

25. Gehrie, E., Van der Touw, W., Bromberg, J. S. & Ochando, J. C. Plasmacytoid dendritic cells in tolerance. *Methods Mol Biol* **677**, 127–147 (2011).

26. Oracki, S. A., Walker, J. A., Hibbs, M. L., Corcoran, L. M. & Tarlinton, D. M. Plasma cell development and survival. *Immunol Rev* **237**, 140–159 (2010).

27. Venkataraman, C., Shankar, G., Sen, G. & Bondada, S. Bacterial lipopolysaccharide induced B cell activation is mediated via a phosphatidylinositol 3-kinase dependent signaling pathway. *Immunol Lett* **69**, 233–238 (1999).
