# Supplementary material for: Early human fetal lung atlas reveals the temporal dynamics of epithelial cell plasticity
Source: Nat Commun. 2024 Jul 13;15:5898. doi: 10.1038/s41467-024-50281-5 (PMC11246468; doi:10.1038/s41467-024-50281-5)
Supplement: Supplementary file 3 — Description of additional supplementary files [file 41467_2024_50281_MOESM3_ESM.pdf]

<u>Description of Additional Supplementary files</u>

Supplementary Data 1: List of cell type markers top differentially expressed genes calculated using Wilcoxon rank sum test.

Supplementary Data 2: Percentages of cell subtypes.
