# Supplementary material for: Early human fetal lung atlas reveals the temporal dynamics of epithelial cell plasticity
Source: Nat Commun. 2024 Jul 13;15:5898. doi: 10.1038/s41467-024-50281-5 (PMC11246468; doi:10.1038/s41467-024-50281-5)
Supplement: Supplementary file 6 — Reporting Summary [file 41467_2024_50281_MOESM6_ESM.pdf]

Corresponding author(s): Dr. Amy P. Wong

Last updated by author(s): May 23, 2024

Reporting Summary

Nature Portfolio wishes to improve the reproducibility of the work that we publish. This form provides structure for consistency and transparency in reporting. For further information on Nature Portfolio policies, see our Editorial Policies and the Editorial Policy Checklist.

Statistics

For all statistical analyses, confirm that the following items are present in the figure legend, table legend, main text, or Methods section.

n/a	Confirmed	
☐	☒	The exact sample size (n) for each experimental group/condition, given as a discrete number and unit of measurement
☒	☐	A statement on whether measurements were taken from distinct samples or whether the same sample was measured repeatedly
☐	☒	The statistical test(s) used AND whether they are one- or two-sided<br>*Only common tests should be described solely by name; describe more complex techniques in the Methods section.*
☐	☒	A description of all covariates tested
☐	☒	A description of any assumptions or corrections, such as tests of normality and adjustment for multiple comparisons
☐	☒	A full description of the statistical parameters including central tendency (e.g. means) or other basic estimates (e.g. regression coefficient) AND variation (e.g. standard deviation) or associated estimates of uncertainty (e.g. confidence intervals)
☐	☒	For null hypothesis testing, the test statistic (e.g. F, t, r) with confidence intervals, effect sizes, degrees of freedom and P value noted<br>*Give P values as exact values whenever suitable.*
☐	☒	For Bayesian analysis, information on the choice of priors and Markov chain Monte Carlo settings
☒	☐	For hierarchical and complex designs, identification of the appropriate level for tests and full reporting of outcomes
☐	☒	Estimates of effect sizes (e.g. Cohen's d, Pearson's r), indicating how they were calculated

Our web collection on statistics for biologists contains articles on many of the points above.

Software and code

Policy information about availability of computer code

Data collection

The cDNA were prepared with the 10X Chromium Next GEM single-cell 3′ Reagent Kits v3.1 (10x Genomics, cat#1000121, 1000120, 1000123) and sequenced using the Illumina NovaSeq6000 sequencer. Reads were aligned to the human reference genome (hg 19, GRCh38) and libraries were demultiplexed and aligned using 10X Cell Ranger v6.0.1. Seurat v4.0 was used for subsequent analysis. For spatial transcriptomics, tissue sections were generated and stained with hematoxylin & eosin for 10X Visium platform (FFPE v2) analyses. The Visium Human Transcriptome Probe Set v2.0 was used. Spaceranger (2.1.0) was used to perform demultiplexing and alignment to the GRCh38-2020-A reference. For 10x Xenium in situ analysis. we created a custom panel (CFVU2E) building off the pre-designed Xenium Human Lung Gene Expression Panel (cat #: 1000601).
Immunofluorescence images were obtained using the Quorum Spinning Disc Confocal microscope equipped with a Hamamatsu C9100-13 EM-CCD camera and 10x/0.4, 20x/0.75, 40x/0.95 and 60x/1.35 (water) objectives. Captured images were analyzed using the Velocity software.
Human pluripotent stem cell (PSC) lines CA1 (courtesy of Dr. Andras Nagy, Lunenfeld-Tenanbaum Research Institute, Toronto) and BU3 (courtesy of Dr. Darrell Kotton, Boston University) were differentiated into fetal lung phenotypes using our directed differentiation protocol. Human PSC-derived fetal lung cells and organoids represent to early and later stages along the differentiation process. Technical replicates (N=3 independant differentiation sets) were pooled to create the single cell libraries for sequencing using the 10X platform as above.

Data analysis

For the scRNA-seq analyses, we used Cell Ranger v6.0.1 and the following R and Python packages: Seurat (v4.0), SeuratData (v0.2.2), SCENIC (v1.3.1), SCopeLoomR (v0.13.0), clustree (v0.5.0), enRichr (v3.2), slingshot (v2.7.0), tidyverse (v2.0.0), viridis (v0.6.2), grDevices (v4.2.1), Matrix (v1.6.4/1.6.5), rgl (v1.2.3), clusterExperiment (v2.16.0), gam (v1.22.2), AUCell (v1.18.1), foreach (v1.5.2), data.table (v1.14.1), pheatmap (v1.0.12), gplots (v3.1.3), SingleCellExperiment (v1.16.0), tradeSeq (v1.8.0), ggplot2 (v3.5.0), CellChat (v1.6.1), numpy (1.22.4/1.23.5), pandas (1.4.2, 2.0.3), scanpy (1.9.1/1.10.1), scvelo (0.2.5), cellrank (2.0.0), anndata (0.8.0), matplotlib (3.5.2), sklearn (0.24.0), seaborn (0.13.0/0.13.2), squidpy (1.2.3), scFates (1.0.6), loompy (3.0.6), MulticoreTSNE (0.1).

Codes for the computational analyses are available at https://github.com/spencerfar/fetal_lung_analysis (DOI: 10.5281/zenodo.11231149) and https://github.com/The-HQQ/Human_fetal_lung_atlas/ (DOI: 10.5281/zenodo.11238128) respectively.

For manuscripts utilizing custom algorithms or software that are central to the research but not yet described in published literature, software must be made available to editors and reviewers. We strongly encourage code deposition in a community repository (e.g. GitHub). See the Nature Portfolio guidelines for submitting code & software for further information.

Data

Policy information about availability of data

All manuscripts must include a data availability statement. This statement should provide the following information, where applicable:

- Accession codes, unique identifiers, or web links for publicly available datasets
- A description of any restrictions on data availability
- For clinical datasets or third party data, please ensure that the statement adheres to our policy

The single cell RNA sequencing and spatial sequencing data generated in this study have been deposited in the NCBI GEO database under accession codes GSE264398,
GSE264425, GSE264407, GSE266789. The raw sequencing files can be accessed on the NCBI website through the accession codes and all processed sequencing data can be obtained at Synapse (https://www.synapse.org/#!Synapse:syn53437291/files/). The source data generated in this study are available at https://www.synapse.org/#!Synapse:syn59808546. The published human fetal lung datasets were obtained from He et al. (Array Express; E-MTAB-11278), Sountoulidis et al. (GEO; GSE215898), and Cao et al. (OMIX; OMIX003147) and mouse embryonic dataset obtained from Negretti et al. (GEO; GSE165063)
The adult lung single cell RNA sequencing dataset was obtained from the Human Cell Atlas portal
(https://datasets.cellxgene.cziscience.com/2aa90e63-9a6d-444d-8343-8fc2a9921797.rds).

Research involving human participants, their data, or biological material

Policy information about studies with human participants or human data. See also policy information about sex, gender (identity/presentation), and sexual orientation and race, ethnicity and racism.

Reporting on sex and gender	No tissues collected were excluded. We collected both male and female lung tissues for our sequencing analyses. The sex of the tissue was determined retrospectively through analysis of the expression of sex-determining genes SRY and XIST. To our knowledge, there are no sex or gender effects that influences the outcome of our analysis especially in this particularly early developmental stage of fetal development. As such, we did not perform sex-based analysis as we did not obtain more than one lung samples for some gestational weeks.
Reporting on race, ethnicity, or other socially relevant groupings	Not applicable.
Population characteristics	For this study, we did not collect the population characteristics of the pregnant women. Only tissues from "normal" or "healthy" pregnancies were collected by the Biobank. No other exclusion criteria related to the mother's medical condition or the fetus were used. There was no bias in tissue collection.
Recruitment	All tissue collections were obtained from the Mount Sinai Hospital Research Centre for Women's and Infants' Health (RCWIH) biobank with REB approval from both the Mount Sinai Hospital Research Ethics Board and the Hospital for Sick Children Research Ethics Board.
Ethics oversight	Human fetal lung tissue collection for the purpose of understanding human lung cell development was obtained from the Research Centre for Women's and Infants' Health (RCWIH) biobank using a protocol in accordance and approved by the Mount Sinai Hospital Research Ethics Board (REB #: 20-0035-E, March 2024) and the Hospital for Sick Children Research Ethics Board (REB #: 1000067499, January 2024). Tissue collection for research use was approved for gestational week 10-20 lung tissues. Fetal lung tissues were harvested from voluntary elective pregnancy terminations (up to 20 weeks gestation) where consent was obtained for the donation of fetal tissues for research.<br><br>The study protocol for use of human pluripotent stem cells (CA1 and BU3, female and male lines respectively) for in-vitro differentiations into lung cell lineages were approved by the Canadian Institutes of Health Research Stem Cell Oversight Committee and Hospital for Sick Children Research Ethics Board (REB #: 1000071246, June 2023).

Note that full information on the approval of the study protocol must also be provided in the manuscript.

Field-specific reporting

Please select the one below that is the best fit for your research. If you are not sure, read the appropriate sections before making your selection.

☒ Life sciences ☐ Behavioural & social sciences ☐ Ecological, evolutionary & environmental sciences

For a reference copy of the document with all sections, see nature.com/documents/nr-reporting-summary-flat.pdf

Life sciences study design

All studies must disclose on these points even when the disclosure is negative.

Sample size	No statistical method was used to predetermine sample size.
Data exclusions	Cells with less than 200 features, greater than 15% mitochondrial transcript, and genes expressed in less than 3 cells were excluded from our analysis. Furthermore, principal component analysis (PCA) for each sample was done to ensure filtering of doublets and high-quality cells. Gene expression levels were log normalized datasets and highly variable features that identify high cell-cell variation within each sample dataset were determined using Seurat's FindVariableFeatures. Regarding all other analyses, no data were excluded.
Replication	All attempts to repeat experiments were successful. For the same gestational age lungs, where multiple donors were available, our analyses pipeline showed reproducible results.<br>For added level of reproducibility, we leverage multiple computational teams to perform the computational analyses. We used multiple methods in our analyses to validate the results and we sourced computational support from the SickKids Computational medicine team (three of them coauthors on this paper) as well as the two co-first authors who performed most of the computational analyses are from different labs to perform the analyses independantly to ensure reproducibility of the findings.
Randomization	For some of the analysis, we grouped samples based on gestational age to represent 3 developmental time points. This enabled us to increase the sample size per group. This method and the rationale for doing so is explained in the manuscript.
Blinding	The investigators were not blinded in the data collection or analysis. This was not applicable in this study due to sample size limitations.

Reporting for specific materials, systems and methods

We require information from authors about some types of materials, experimental systems and methods used in many studies. Here, indicate whether each material, system or method listed is relevant to your study. If you are not sure if a list item applies to your research, read the appropriate section before selecting a response.

Materials & experimental systems

n/a	Involved in the study	
☐	☒	Antibodies
☒	☐	Eukaryotic cell lines
☒	☐	Palaeontology and archaeology
☒	☐	Animals and other organisms
☒	☐	Clinical data
☒	☐	Dual use research of concern
☒	☐	Plants

Methods

n/a	Involved in the study	
☒	☐	ChIP-seq
☒	☐	Flow cytometry
☒	☐	MRI-based neuroimaging

Antibodies

Antibodies used

All primary and secondary antibodies used in this study is listed in details in Supplementary Table 3. Antibodies are listed by protein target, host specie of antibody, supplier, catalog number and, working dilution

Primary antibodies

anti-ACTA2, Rabbit, Abcam, ab124964, 1:500
anti-ASCL1, Goat, Rockland, 600-101-MH4, 1:500
anti-CFTR, Mouse, Millipore, MAB1660, 1:70
anti-Delta NP63, Mouse, Abcam, ab735, 1:100
anti-Desmin, Goat, R&D, AF3844-SP, 1:100
anti-FOXJ1, Rabbit, Abcam, ab235445, 1:100
anti-MEF2C, Mouse, Invitrogen, MA5-25477, 1:50
anti-NKX2-1, Rabbit, Abcam, ab76013, 1:200
anti-SCGB3A2, Goat, Santa Cruz, sc-48320, 1:50
anti-SFTPB, Rabbit, Abcam, ab40876, 1:50
anti-SOX2, Goat, R&D, AF2018, 1:300
anti-SOX9, Goat, R&D ,AF3075, 1:600

Secondary Antibodies

Anti-mouse IgG (H+L) Alexa Fluor 488, Donkey, Invitrogen, A21202, 1:500
Anti-rabbit IgG (H+L) Alexa Fluor 488, Donkey, Invitrogen, A21206, 1:500
Anti-rat IgG (H+L) Alexa Fluor 488 ,Donkey, Invitrogen, A21208, 1:500
Anti-mouse IgG (H+L) Alexa Fluor 546, Donkey, Invitrogen, A10036, 1:500
Anti-rabbit IgG (H+L) Alexa Fluor 546, Donkey, Invitrogen, A10040, 1:500
Anti-goat IgG (H+L) Alexa Fluor 546, Donkey, Invitrogen, A11056, 1:500

Anti-rat IgG (H+L) Alexa Fluor 555, Donkey, Invitrogen, A48270, 1:500
Anti-Rabbit IgG (H&L) Alexa Fluor 647, Donkey, Abcam, ab150075, 1:500
Anti-goat IgG (H+L) Alexa Fluor 647, Donkey, Invitrogen,A21447, 1:500

Validation

For validation of all primary antibodies, the manufacturer provides information regarding the species reactivity and the recommended concentrations in relevant citations. For each antibody, we also perform a titration analysis based on the recommended concentrations to determine the optimal concentration for best signal to noise outcome in the staining. Negative controls were done using non-immune isotype controls or "no primary" controls on serial sections of the lung tissue.

Plants

Seed stocks

Report on the source of all seed stocks or other plant material used. If applicable, state the seed stock centre and catalogue number. If plant specimens were collected from the field, describe the collection location, date and sampling procedures.

Novel plant genotypes

Describe the methods by which all novel plant genotypes were produced. This includes those generated by transgenic approaches, gene editing, chemical/radiation-based mutagenesis and hybridization. For transgenic lines, describe the transformation method, the number of independent lines analyzed and the generation upon which experiments were performed. For gene-edited lines, describe the editor used, the endogenous sequence targeted for editing, the targeting guide RNA sequence (if applicable) and how the editor was applied.

Authentication

Describe any authentication procedures for each seed stock used or novel genotype generated. Describe any experiments used to assess the effect of a mutation and, where applicable, how potential secondary effects (e.g. second site T-DNA insertions, mosiacism, off-target gene editing) were examined.
